# Take time: odor coding capacity across sensory neurons increases over time in *Drosophila*

**DOI:** 10.1007/s00359-017-1209-1

**Published:** 2017-08-29

**Authors:** Daniel Münch, C. Giovanni Galizia

**Affiliations:** 1 Neurobiology, University of Konstanz, 78457 Konstanz, Germany; 2 Present Address: Champalimaud Neuroscience Programme, Champalimaud Centre for the Unknown, Av. Brasília, 1400-038 Lisbon, Portugal

**Keywords:** Combinatorial code, Temporal code, Adaptation, Odor mixture, Olfaction

## Abstract

Due to the highly efficient olfactory code, olfactory sensory systems are able to reliably encode enormous numbers of olfactory stimuli. The olfactory code consists of combinatorial activation patterns across sensory neurons, thus its capacity exceeds the number of involved classes of sensory neurons by a manifold. Activation patterns are not static but vary over time, caused by the temporally complex response dynamics of the individual sensory neuron responses. We systematically analyzed the temporal dynamics of olfactory sensory neuron responses to a diverse set of odorants. We find that response dynamics depend on the combination of sensory neuron and odorant and that information about odorant identity can be extracted from the time course of the response. We also show that new response dynamics can arise when mixing two odorants. Our data show that temporal dynamics of odorant responses are able to significantly enhance the coding capacity of olfactory sensory systems.

**Electronic supplementary material:**

The online version of this article (doi:10.1007/s00359-017-1209-1) contains supplementary material, which is available to authorized users.

## Introduction

Neural networks increase their coding capacity using combinatorial logic, rather than coding information in single neurons (the “grandmother cells”), information resides in activity patterns across many neurons. Olfactory coding is a powerful example for such an approach, humans have approximately 350 sensory neuron classes (OSN), but can smell thousands of substances, fruit flies with approximately 50 OSN classes (Couto et al. 2005; Benton et al. 2009; Grabe et al. 2015) are similarly powerful. At very low concentrations, a substance might only activate a single class of OSNs: in this range, with 50 receptors, the capacity of the system is 50 odors. However, as concentration increases, more than one OSN class is activated, and the capacity of the system increases exponentially. The *Drosophila* olfactory sensory system has been analyzed to a great extent, with good examples for information carried by single OSN classes (Kwon et al. 2007; Stensmyr et al. 2012; Dweck et al. 2015), and extensive analyses of combinatorial coding patterns (Hallem and Carlson 2006; Galizia et al. 2010; Campbell et al. 2013; Münch and Galizia 2016).

With a binary code, 50 OSN classes would allow for 2^50^ patterns, i.e., 10^15^ patterns (Galizia 2014). However, since the activation pattern also changes with odorant concentration, one odor needs more than one pattern, thus reducing the capacity of the system. On the other hand, OSN responses are not binary (on/off), but continuous (weak responses, strong responses), leading to an increase in the information capacity (de Bruyne et al. 2001; Hallem and Carlson 2006). Indeed, single OSNs can encode more than one odorant, probably exploiting the time course of an odorant response (DasGupta and Waddell 2008). Many more factors add complexity here, OSNs may be in an adapted state when hit by an odor plume (de Bruyne et al. 1999), and odor plumes may be temporally complex (Murlis et al. 2000; Szyszka et al. 2014). Furthermore, the chemical space of odors to be coded is not limited to the many chemical substances, since odors are generally not single chemical substances. Rather, odors are generally mixtures of substances, with varying ratios of their key components in an ecological setting (Jordán et al. 2001; Locatelli et al. 2016).

**Fig. 1:**
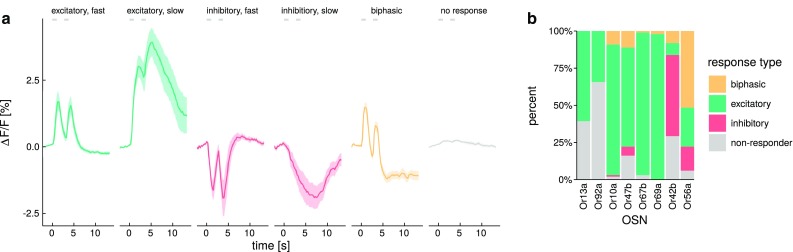
Response dynamics are temporally diverse. All odorants given at 10$^{-2}$ dilution. **a** The different response dynamics we observed could be grouped into four different categories. Excitatory and inhibitory responses could be further subdivided into “fast” and “slow” responses. Traces are given as average of *n* = 4–10 animals, *shades* indicate SEM. *Gray* segments indicate the stimulation times. **b** The four main response types were differentially distributed across OSN classes. Response types were automatically defined, responses with maxima below a threshold of $|0.3\%|\Delta F/F$ ($\pm 2.5\times {\text {SD}}$ before stimulus onset) were defined as “non-responders” (see “Material and methods” for details)

For this reason, we have sought to analyze the combinatorial odorant response code in a systematic way, using *Drosophila melanogaster* as a model system. We have measured the responses to 99 odorants in eight different classes of olfactory sensory neurons, both in a non-adapted and an olfactory-adapted state. Furthermore, we have systematically studied the responses to odorant mixtures. We found that responses show complex but reproducible temporal trajectories, which greatly add to the coding capacity of the system. We hypothesize that animals may respond fast to the initial activity pattern evoked by an olfactory stimulus, but then may have the capacity to fine-tune their sensory analysis. Such a two-step odorant evaluation behavior needs to be tested experimentally.

## Materials and methods

### Animals

All recordings were performed on female *Drosophila melanogaster* expressing the calcium reporter GCaMP 1.3 (Nakai et al. 2001) or GCaMP 3 (Tian et al. 2009) under the control of the GAL4-UAS expression system. UAS-GCaMP 1.3 flies were provided by Jing Wang, University of California, San Diego, La Jolla, CA; UAS-GCaMP 3.0 flies were provided by Loren L. Looger, Howard Hughes Medical Institute, Janelia Farm Research Campus, Ashburn, Virginia. Stable GAL4-UAS fly lines were of the following genotypes: P[UAS:GCaMP1.3]; P[GAL4:X] (X being one of Or10a, Or13a, Or22a, Or42b, Or47a, Or47b, Or67b, Or69a or Or92a), and P[Or56a:GAL4]; P[UAS:GCaMP3]attP40. Flies were kept at 25 $^{\circ }\mathrm{C}$ in a 12/12 light/dark cycle at 60–70% RH. Animals were reared on standard medium (100 mL contain: 2.2 g yeast, 11.8 g of sugar beet syrup, 0.9 g of agar, 5.5 g of cornmeal, 1 g of coarse cornmeal and 0.5 mL of propionic acid).

### Odorants

Odorants were purchased from Sigma-Aldrich in the highest purity available. Pure substances were covered with Argon to avoid oxidation. Odorants were prepared as dilutions (ranging from 10$^{-2}$ to 10$^{-6}$) in 5 mL mineral oil (Sigma-Aldrich, Steinheim, Germany). Odorants were prepared as 5 mL diluted substances in 20 mL head space vials covered with pure nitrogen to avoid oxidation (Sauerstoffwerk Friedrichshafen GmbH, Friedrichshafen, Germany) and immediately sealed with a Teflon-coated septum (Axel Semrau, Germany). Single odorants were applied at 10$^{-2}$ dilution, mixture components at 10$^{-3}$. Concentration series were recorded at dilutions ranging from 10$^{-2}$ to 10$^{-6}$. A complete list of odorants is given in Table S1.

### Calcium imaging

Calcium imaging was performed on either of two setups which consisted of a fluorescence microscope (BX50WI or BX51WI, Olympus, Tokyo, Japan) equipped with a $50\times$ air lens without cover slip correction (Olympus LM Plan FI $50{\times }/0.5$). Images were recorded with a CCD camera (SensiCam, PCO, Kelheim, Germany) with $8\times 8$ pixel on-chip binning, which resulted in $80\times 60$ pixel-sized images. We recorded each stimulus for 20 s at a rate of 4 Hz using TILLvisION (TILL Photonics, Gräfelfing, Germany). A monochromator (Polychrome II or Polychrome V, TILL Photonics, Gräfelfing, Germany) produced excitation light of 470 nm wavelength which was directed onto the antenna *via* a 500 nm low-pass filter and a 495 nm dichroic mirror, emission light was filtered through a 505 nm high-pass emission filter. We recorded between 7 and 98 odorant responses per animal (median = 35).

### Stimulus application

A computer-controlled gas chromatography autosampler (PAL, CTC Switzerland) was modified and used for automatic odorant application. A head space of 2 mL from the 20 mL vials was injected into a continuous flow (60 mL min$^{-1}$) of purified air in two 1 mL portions spaced by 2.75 s with an injection speed of 1 mL s$^{-1}$. This procedure further diluted the stimulus 1:2. Stimuli arrived at the antenna with setup-specific delays, therefore, the first stimulus onset was defined at *t* = 0. The stimulus was directed at the antenna of the animal *via* a Teflon tube (inner diameter 1 mm, length 39.5 cm, with the exit positioned ${\sim }$2 mm from the antenna). Stimulus properties were measured with a photoionization detector (miniPID, model 200A, Aurora Scientific, Ireland). Blocks of four to eight odorants were presented (ISI >2 min) interspaced by solvent control, room air control and an OSN-specific reference odorant. The reference odorants were **Or10a**: butyl acetate (InChIKey: DKPFZGUDAPQIHT-UHFFFAOYSA-N), **Or13a**: 3- octanol (NMRPBPVERJPACX-UHFFFAOYSA-N) **Or22a**: ethyl propionate (FKRCODPIKNYEAC-UHFFFAOYSA-N), **Or42b**: ethyl propionate (FKRCODPIKNYEAC-UHFFFAOYSA-N), **Or47**a: hexyl acetate (AOGQPLXWSUTHQB-UHFFFAOYSA-N), **Or47b**: (S)-(+)-carvone (ULDHMXUKGWMISQ-VIFPVBQESA-N), **Or56a**: 2-hexanol (QNVRIHYSUZMSGM-UHFFFAOYSA-N), **Or67b: **1-hexanol(ZSIAUFGUXNUGDI-UHFFFAOYSA-N), **Or69a**: isopentanoic acid (GWYFCOCPABKNJV-UHFFFAOYSA-N), **Or92a**: 2,3-butanedione (QSJXEFYPDANLFS-UHFFFAOYSA-N). After each injection, the auto sampler syringe was flushed with purified air for 30 s. After each block of stimuli, the syringe was washed with hexane or pentane (Merck, Darmstadt, Germany), heated up to 48 $^{\circ }\mathrm{C}$, and flushed with continuous clean air stream for ${\sim }$6 min.

### Mixture application

Mixture application was performed as above but using two computer-controlled autosamplers (Twin-PAL, CTC Switzerland). Injections were essentially performed as described above but the two components of each mixture were injected simultaneously. The two autosamplers injected into two separate arms of a y-shaped Teflon tube (inner diameter 2 mm, length 47.5 mm) with an injection speed of 1 mL s$^{-1}$. The combined air stream was directed onto the antenna of the fly *via* the outlet of the y-tube. Using two modified autosamplers, it was possible to perform mixture experiments without pre-mixed chemicals, excluding molecular interactions of ligands in solution and possible influence on individual headspace concentrations. Both components were injected at the same time, creating an “on the fly” mixture within the stimulus tube. Blocks of five stimuli were presented interspaced by controls as described above. Odorants to be mixed were selected on the basis of their temporal response profiles, to analyze a range of different combinations. Odor vials were labeled with barcodes containing odor and concentration information. Barcodes were scanned and recorded by the autosampler system after each stimulation.

**Fig. 2:**
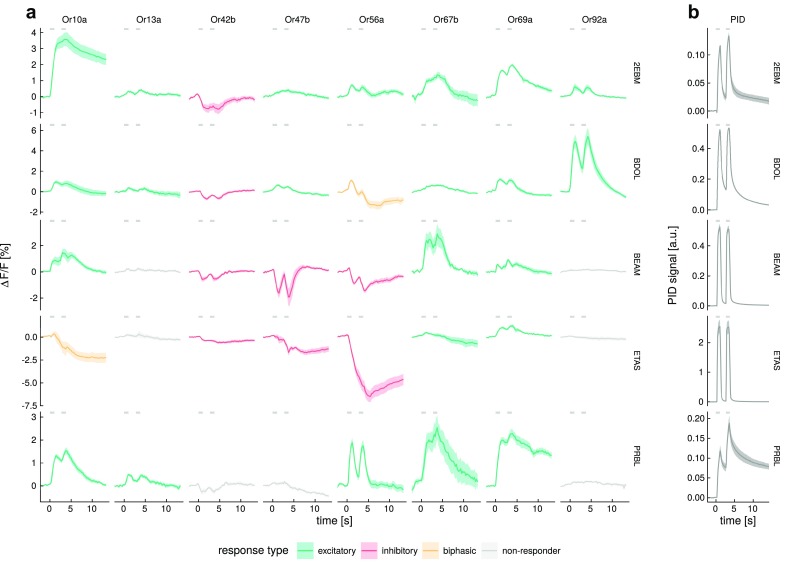
Response dynamics depend on the odorant–OSN combination. **a** Example calcium imaging response traces for eight OSNs stimulated with five odorants. Traces are given as average of *n* = 4–13 animals, shades indicate SEM, colors indicate response type as in Fig. 1. **b** PID measurements of the stimulus dynamics for the five example odorants. Traces are given as average of *n* = 1–3 independent measurements. See Fig. S2 for response traces and PID measurements of all 99 odorants. For a list of odorant abbreviations see Table S1. *Gray* segments indicate the stimulation times

### Data analysis

We analyzed calcium imaging data using custom written routines in IDL (ITT VIS, USA) and Gnu-R (R Core Team 2017). Recorded movies were manually corrected for lateral movement artifacts. Then, an area of interest was defined for the parts of the antenna that showed fluorescence increase upon stimulation. Time traces were averaged across this area. We included all measurements into the analysis as long as animals showed stable responses to the reference odorant. Relative percentage fluorescence change was calculated as $\Delta F/F=((F_{i}-F_{0})/F_{0})\times 100$ with $F_{i}$ being the fluorescence at $frame_{i}$ and $F_{0}$ being the mean fluorescence of 5 s before stimulus onset. To correct for the photo bleaching of the dye, we fitted an exponential decay function of the form $A \times exp^{-x/B}+C$ to each response trace using the nls() function in *R*. Because some odorant responses would not reach baseline within measurement time, the decay rate parameter *B* was estimated from the median mineral oil control trace within each animal. We omitted 750 ms at the beginning of the time-trace and 11 s during the response. The pre-stimulus part of the recording was weighted 100 fold (Galizia and Vetter 2004). We corrected for calcium signal decrease across measurements, likely due to GCaMP bleaching, using a linear regression across reference odorant measurements within each individual animal. The value of this function at each corresponding time point was used to scale responses using the first reference odorant presentation as reference.

**Fig. 3:**
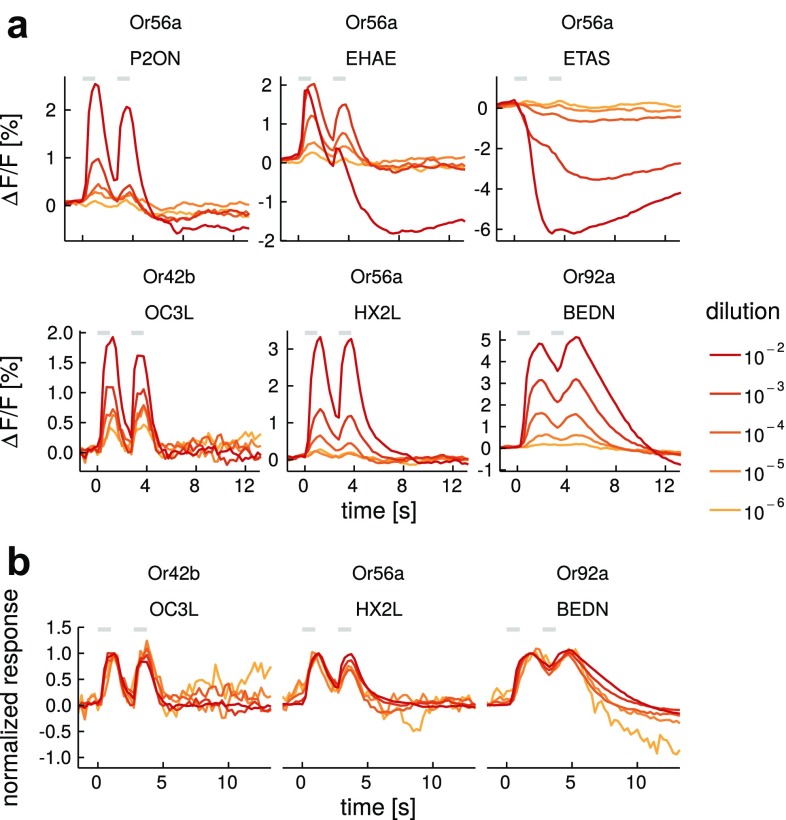
Response dynamics are stable across a concentration range. **a** Recordings of five odorant–OSN combinations at five dilution steps. Traces are given as average of *n* = 3–17 animals. *Colors* indicate different dilutions. **b** Same recordings as in the *lower panel* in **a** but normalized to the first response peak. *Gray* segments indicate the stimulation times. For a list of odorant abbreviations see Table S1

#### Response classification

To automatically classify responses to the four different response categories (excitatory, inhibitory, biphasic, and non-responder) we extracted response magnitudes as the mean of five frames around three different time points during the response: peak 1 (1.5 s after stimulus onset), peak 2 (4 s after stimulus onset and post (6.5 s after stimulus onset). We defined ±2.5$\times$ the standard deviation of the activity during 5 s before stimulus onset as a response threshold, which corresponded to $|0.3\%|\Delta F/F$. All responses that did not exceed the response threshold at any of the three time windows were classified as non-responders, remaining responses were classified as responders accordingly. Responders were classified as biphasic when response values at peak 1 were positive and at post were negative. Remaining responses were classified excitatory when the response value at peak 1 was positive and inhibitory when activity at peak 1 was negative. Classifications were performed on mean response traces with n ranging from 3 to 28 with a median of 8 flies.

#### Bootstrapping

We recorded calcium imaging data from flies that expressed a calcium reporter in a single class of OSN. We used a bootstrapping-like approach to derive ensemble response patterns from this data. From the entity of recordings (99 odorants $\times$ 8 OSNs $\times$ *n* of 3–28) we randomly sampled recordings to create “meta animals” that consisted of a complete set of odorant $\times$ OSN combinations.

#### Odorant discriminability

To quantify the difference between odorant activity patterns, we created 100 “meta animals” (as described above), yielding 100 coordinates per odorant in an eight-dimensional OSN space. We measured odorant pattern distance as Euclidean distance between the centroids of the 100 repetitions per odorant. To obtain a discriminability measure we corrected the distance measure by the variability within the 100 repetitions per odorant. Therefore, we calculated the average distance between the 100 coordinates per odorant to their respective centroid. Finally, we divided the distance between the centroids of each odorant pair by the sum of the corresponding intra-odorant variabilites.With *n* = 100 fictive responses A$_\mathrm{i}$ to odorant *A* and *B* $_\mathrm{i}$ to odorant *B*, this gives:

$$\begin{aligned} \frac{\Vert {\text {centroid}}_{A}-{\text {centroid}}_{B}\Vert }{{ \frac{1}{n}\sum \nolimits _{i=1}^{n}\Vert A_{i}-{\text {centroid}}_{A}\Vert +\frac{1}{n}\sum \nolimits _{i=1}^{n}\Vert B_{i}-{\text {centroid}}_{B}\Vert }} \end{aligned}$$

#### Odorant classification

To quantify odorant identity information at different time points during the odor response, we trained a linear classifier (linear discriminant analysis using the lda function in R) on a subset of the data and tested its performance on a different subset. In detail: (1) We created a test dataset by sampling one “meta animal” (as described above), these data were removed from the pool of recordings. (2) From the remaining data we sampled a training dataset consisting of ten “meta animals”. (3) We used the training data to train the classifier on different time points of the responses. (4) We then used the trained classifier to predict the correct labels of the 99 odorant responses from the test dataset and noted its performance. (5) We repeated steps 1–4 1000$\times$ to get estimates of the average performance and variability of the classification. We performed the classification analysis on the complete set of 99 odorants as well as on smaller subsets. For the subsets, we ranked odorants according to the overall response strength they elicited across OSNs and divided them into four sets. To create equal-sized sets of 25 odorants each, one odorant was assigned both to set 3 and 4. To obtain the ranking, for each response we calculated the absolute peak response after stimulation and averaged across OSNs per odorant. See Table S2 for the obtained ranking.

**Fig. 4:**
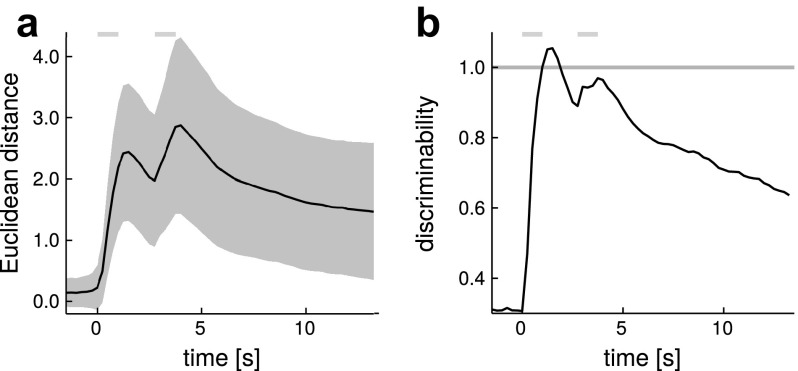
Odorant pattern difference peaks during stimulation. **a** The average Euclidean distances between response patterns of all possible odorant pairs. *Shades* indicate the variability within the repeated measurements of a given odorant (see “Material and methods” for details), *gray* segments indicate the stimulation times. **b** Average discriminability of all possible pairs of odorants. The discriminability measure was derived from dividing Euclidean distances by the variability within repeated measurements of an odorant (see “Material and methods” for details). *Gray* shades indicate SEM, *gray* segments indicate the stimulation times

#### Mixture trajectories

For each binary mixture, we performed a principal component analysis (PCA) on the peak response of the component responses using the prcomp function in R. Thereby the PCA was optimized to separate the components’ peak responses. The resulting rotation was then applied to the full response traces including the mixture trace. For better visibility, we removed the initial part of the recording till 1 s prior to the first stimulation in Figs. 6b and S7.

#### Statistical testing

All statistical testing was performed in R using base functions and the PMCMR package.

## Results

### Complex response dynamics of OSNs

We recorded calcium responses from eight different classes of *Drosophila *olfactory sensory neurons (OSN) in response to stimulation with 99 mono-molecular odorants. Response magnitudes differed widely, ranging from −6.6 to 5.9% changes in $\Delta F/F$. Some responses were excitatory (calcium increases, green traces in Fig. 1a), some were inhibitory (calcium decreases, red traces in Fig. 1a), some odorants elicited biphasic responses (yellow trace in Fig. 1a), and some did not elicit any response (gray trace in Fig. 1a; see Fig. S1 for histograms of the classified responses). Biphasic responses were always excitatory first, and inhibitory later—we never observed the inverted sequence. Biphasic responses were strongest at high odorant concentrations. We found large differences in response shape over time: phasic responses were fast, and returned to baseline within a few seconds, while slow responses had a slow return to baseline, generally not complete within the 15 s recording time. Since our stimuli were always 1 s, we did not evaluate whether some responses had a tonic component, i.e., whether calcium concentration would have stabilized to a different value with ongoing stimulation. Our stimulus protocol consisted in two 1 s stimuli, spaced by 2.75 s. We found that the response to the second stimulus, hitting a weakly adapted OSN, was clearly visible in most cases. For fast responses, the second stimulus generally elicited a response of similar shape and size as the first stimulus (both in the excitatory and the inhibitory case), indicating that no adaptation had remained (see, e.g., first and third trace in Fig. 1a). For slow responses, the second stimulus elicited a response riding on top of the first one, leading to a stronger overall response, even though the response to the second stimulus alone was smaller (see e.g., second trace in Fig. 1a). In many cases, slow responders did not show any visible response to the second stimulus, suggesting a completely adapted OSN, but we cannot exclude that the second stimulus might have augmented the return to baseline time constant (Fig. 1a).

### Response categories are differentially distributed across OSN classes

We performed an automatic classification of the odorant responses into the four main categories of responses we found (no response, excitation, inhibition, biphasic). We defined a response threshold of 0 ± 2.5$\times$ the standard deviation of a 5-s response window before stimulus presentation, averaged across all OSNs and odors. This yielded a threshold of $|0.3|\Delta F/F$, responses that remained within that range were classified as non-responders. We found that for our set of 99 odorants, the response types were differentially distributed across OSN classes. Excitatory responses were the most frequent ones: Or13a and Or92a only showed excitatory responses (or no responses), while Or10a, Or47b, Or67b, and Or69a mainly responded with excitation. Conversely, Or42b exhibited mostly inhibitory and Or56a mainly biphasic responses (Fig. 1b).

### Response dynamics depend on the odorant–OSN combination

Response dynamics did not depend on either the odorant or the OSN alone, but rather on the odorant–OSN combination (Fig. 2). This means that a given odorant was able to elicit different response dynamics across OSNs, and that OSNs were able to respond with different dynamics to different odorants. In addition, responses of OSNs that only showed excitation (and no response, Or67b and Or92a, Fig. 1b) displayed odorant-dependent time-courses (Fig. 2). Most importantly, a given odorant–OSN combination reliably elicited the same type of response (see the error shades along the response traces in Fig. 2). As an example an $odorant\times OSN$ response matrix is shown in Fig. 2 (see Fig. S2 for all odorant–OSN combinations). Temporal diversity could also derive from adsorption and desorption differences of the chemical substances in the stimulus delivery device, therefore, we characterized stimulus dynamics for all odorants with a photoionization detector (PID). We found that indeed different substances generate physically different temporal profiles in their stimulus (Figs. 2, S2). Comparing stimulus and response dynamics demonstrated that both odorants with slow as well as fast stimulus dynamics were able to elicit fast and slow response dynamics (Fig. 2a). For example, BEAM (benzaldehyde) elicited a fast inhibitory response in Or47b returning to baseline between the two stimuli, a slow inhibitory response in Or56a, with the second response riding on top of the first one, and a slow excitatory response to Or67b, even though, physically, the odorant stimulus was very fast (see PID trace; Fig. 2a, b). On the other hand, PRBL ($\gamma$-propyl-$\gamma$-butyrolactone) was a more “sticky” odorant in our olfactometer, with the second PID peak riding on top of the first. Nevertheless, Or56a showed perfectly phasic and fast responses, clearly separating the information of the first and second stimulus peak (Fig. 2a, b). A quantitative analysis of the time courses showed that there was no correlation between the duration of the physical stimulus (PID traces; width at 30% peak response) and the biological response time courses (OSN responses; width at 30% peak response; *r* = 0.06, *p* = 0.13; Fig. S3). For some of the best ligands, we measured concentration series (Fig. 3). Response dynamics were stable across concentrations except for one case where an excitatory response became biphasic in the highest concentration (EHAE [E2-hexenyl acetate] in Fig. 3a). Thus, fast responses stayed fast and slow responses remained slow across concentrations as became clear when normalizing the response to the first response peak (compare lower panel in Fig. 3a, b). We observed signal saturation, i.e., reduced signal at the highest concentration, in 5 out of 50 cases.

**Fig. 5:**
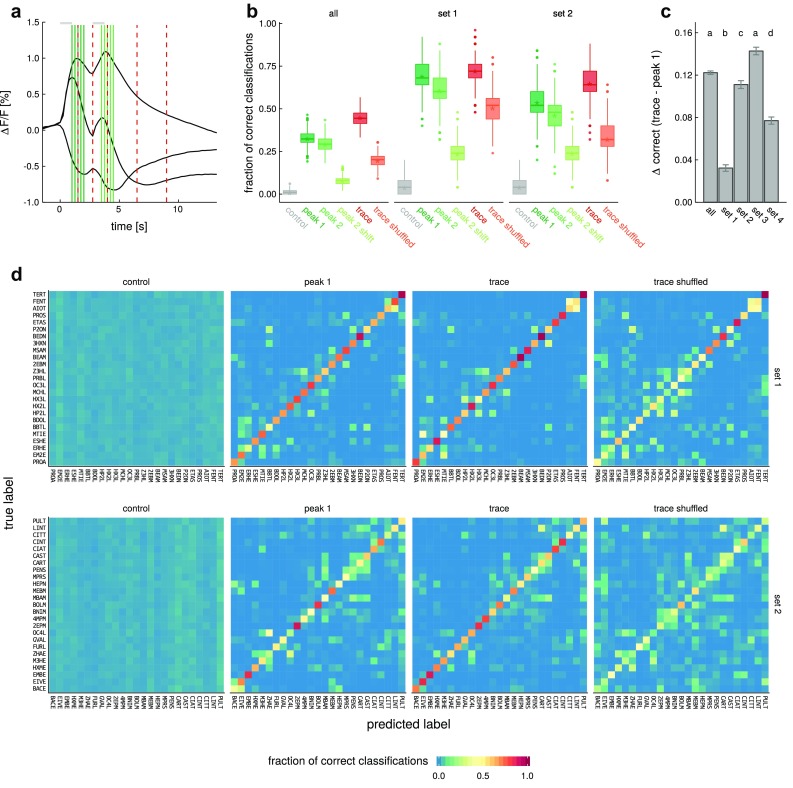
Odorant identity information increases over time. **a** Schematic showing the time points of the recordings that were used for the classification shown in **b** and **d**. *Black* traces are averages across all recordings of a given response type. *Colors* indicate the different time points used in the classification (compare to **b**), *gray* segments indicate the stimulation times. **b** *Boxplot* of the classifier performance at different time points. *control* classification with shuffled odor labels, *peak 1* and *peak 2* five time points around the 1st and 2nd response peak, *peak 2 shift* classification run on peak 2 but with the activity right prior to peak 2 shifted to baseline, *trace* five time points spread across the recording, *trace-shuffled *classification run on the trace frames but with scrambled time information. *all* comprises all odorants, *set 1* contains the top quartile with the 25 strongest odorants (quantified as mean absolute peak response across OSNs),* set 2* contains 2nd best quartile of odorants (see Table S2 for a list of odorants and Fig. S4 for data regarding sets 3 and 4). All differences between classifications at different time points were significant (Kruskal–Wallis rank sum test with a Bonferroni corrected Dunn’s post hoc test, *p* < 0.01). *Boxplots* indicate median, lower, and upper quartile, *whiskers* extend to the lowest and highest values that lie within 1.5 times the inter-quartile range from the *box*, data beyond the *whiskers* are treated as outliers and indicated as *points*, *asterisks* indicate the mean. **c** The differences between the classifier performances at *peak1* and *trace*. *Error bars* indicate SEM. *Different letters* indicate significant differences between groups (Kruskal–Wallis rank sum test with a Bonferroni corrected Dunn’s post hoc test, *p* < 0.01) **d** Correct classifications and classification errors in the sets of the strongest and the weakest odorants at the different time points, visualized as confusion matrices. The values along the diagonal represent classification reliability. See Table S1 for a complete list of odorant names and abbreviations

### Temporal response dynamics carry odorant identity information

Odorant identity is encoded *via* the ensemble response pattern of all the differentially activated OSNs of an olfactory sensory system. Thus, with eight OSNs, we can represent an odor response as a vector of eight dimensions, a vector that changes over time as the response evolves. Before stimulus onset, all measurements will be located close to the origin, reflecting physiological noise. As soon as the odorant responses begin, the representation for each odorant will move to its characteristic place. If two odorants are very different, they will be far away (have a large Euclidean distance) in this eight-dimensional space, if two odorants are similar, they will be close to each other (Sachse et al. 1999; Mazor and Laurent 2005). We calculated the average binary distance of all odorant pairs for each time point of the response (Fig. 4a). The distance increased with the first stimulus, and increased further with the second stimulus, slowly moving towards baseline thereafter. Distance alone, however, is not sufficient for efficient information coding: if single measurements scatter widely (i.e., if noise is high), the brain will not be able to reliably assess the identity of a stimulus. Therefore, we divided the distance between each odorant pair by a quantification of the scattering of each of the individual odorants to visualize general separability (Fig. 4b; see “Material and methods” for details). Values above 1 indicate good average separability between odorant representations, or high information content in the response patterns. While in the raw distance measure, the peak response to the second stimulus was larger than to the first stimulus, the mean discriminability across all odorant pairs peaked during the first stimulation and, though increasing again for the second stimulus, did not reach the same height. It reached a level above 1 only during the first stimulation.

**Fig. 6:**
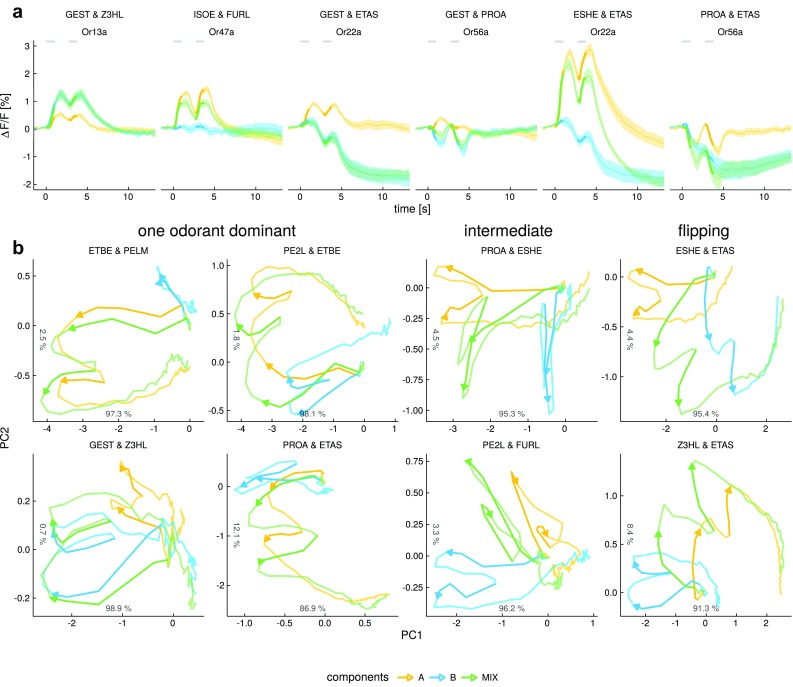
Response dynamics of binary mixtures. **a** Response traces elicited from binary mixtures of odorants. The mixture trace is shown in *green*, the components are shown in *yellow* and *blue*, *gray* segments indicate the stimulation times. Concentration of the components was the same when tested alone or in the mixture ($1\times 10^{-3}\,{\text {vol}}/{\text {vol}}$ dilution). Traces are given as average of *n* = 7–16 animals. **b** Principal component trajectories of mixture and component responses. Trajectories show how the odor response pattern of the five analyzed OSNs develops over time. *Same color* code as in **a**. *Numbers on the axes* indicate the percentage of variance explained by the corresponding principal component. Times of odorant stimulation are indicated by *darker arrows* pointing in the direction of time. See Table S1 for a complete list of odorant names and abbreviations and Figs. S7 and S8 for data of all mixtures tested

The analysis with the Euclidean distance shows that information is present in odorant responses, and peaks during odorant presentation. However, we have seen that temporal response profiles differ. Therefore, we asked whether the brain would benefit from analyzing the whole response time course, or whether the initial, phasic response already contains all relevant odorant information. To this end, we selected different groups of five time points each, and ran a classifier on these. We tested (Fig. 5a): (1) five points at the first response peak (phasic initial response, peak 1), (2) five points at the second response peak (adapted response, peak 2), and (3) five points at the second peak, shifted to a baseline between the two peaks (phasic component of the adapted response). We compared these with (4) five points spread across the response (trace). As controls we shuffled odorant labels (control), or the time information of the trace set (shuffled time). We always selected five time points of the recording to keep the degrees of freedom available to the algorithm comparable.

First, we ran the classifier on the complete set of 99 odorants (Fig. 5b), using bootstrap-like resampling to quantify the reliability of the approach. The algorithm was able to correctly classify a mean of 33% of odorants using peak 1 and 29% based on peak 2. Classification success increased significantly to 45% when the five time points were spread across the recording (trace) (Kruskal–Wallis rank sum test with a Bonferroni corrected Dunn’s post hoc test, *p* < 0.01) and it dropped to 20% when time information was scrambled (Figs. 5b, S6). This demonstrates, that information of odorant identity resides in the temporal dynamics of odorant responses and that using this information improves odorant classification. As expected, the classifier performed on chance level when the odorant identity was shuffled.

We ran the same analysis on subsets of odorants. We ordered the odorants according to response strength by quantifying the mean peak responses an odorant elicited across all eight OSNs and split the 99 odorants set into four subsets (set 1–set 4). The classifier performed better on the smaller sets of odorants except for the set containing the weakest odorants (set 4). Classification success was best for set 1 (the set containing the strongest odorants) and decreased gradually towards set 4 (Figs. S4, S5). The overall classification improved when performed on the *trace* instead of the peak response alone (peak 1) for all sets (Fig. 5c). This was true for all odorant sets as averages, but when looking at the odorants individually, there were cases of individual odorants where classification success went down (Figs. 5d, S5). The improvement in classification was highest for sets 2 and 3, it was low for the set 1 (containing the strongest odorants; Fig. 5c). This demonstrates that information about temporal response dynamics improves odorant identity classification especially for ligands that elicit intermediate responses.

### New response dynamics can arise when mixing odorants

Most olfactory stimuli in the natural world consist of mixtures containing many substances. Responses of olfactory sensory neurons to mixtures have been shown to be complex in several studies (Tabor et al. 2004; Rospars et al. 2008; Hillier and Vickers 2011; Münch et al. 2013). Here, we investigated how odorant mixtures influence the temporal evolution of odorant responses. We used a subset of odorants and OSNs to investigate responses to binary mixtures (Fig. 6a). Apart from changes in response amplitude, mixing two components that both elicited excitatory responses from a given OSN usually resulted in mixture response that resembled the response dynamics of the stronger component. For example, the response of Or13a to the mixture of GEST (geranyl acetate, weak excitatory response) and Z3HL (Z3-hexen-1-ol, excitatory response) resembled the response to Z3HL alone, with no apparent contribution of GEST (Figs. 6a, S7). Or47a responded to the mixture of ISOE (isopentyl acetate, excitatory response) and FURL (furfural, no response), with a reduced response, yet resembling the dynamics of the response to ISOE (Fig. 6a). Dynamics of mixtures containing components that elicited responses of opposing polarity on the other hand were not easily predictable (Figs. 6a, S7). We observed cases where the inhibitory component clearly dominated the mixture response. For example, Or22a responded to the mixture of GEST (geranyl acetate, excitatory response) and ETAS (acetic acid, biphasic response) with a response corresponding to that of ETAS alone, and Or56a responded to the mixture of GEST (geranyl acetate, weak excitatory response) and PROA (propanal, inhibitory response) with a response corresponding to the inhibitory component (Fig. 6a). We found other cases where the excitatory component dominated in the early part of the response, e.g., Or22a responded to the mixture of ESHE (ethyl (*S*)-(+)-3-hydroxybutyrate, strong excitatory response) and ETAS (acetic acid, weak biphasic response) with a strong initial activation followed by an influence of the inhibitory late phase of ETAS that eventually resulted in a strong biphasic mixture response. In other cases again the resulting mixture response was additive, such as in the response of Or56a to PROA (propanal, inhibitory phasic response) and ETAS (acetic acid, inhibitory slow response) (Fig. 6a).

This great variability in mixing logic for individual ORs suggests that the combinatorial response patterns to mixtures are rather configural, i.e., mixture-unique, than elemental. However, this has to be tested in the ensemble response, and not for single OSNs. Therefore, we created response trajectories by calculating response data in the five-dimensional OSN space, and projecting it onto two dimensions. We plotted the mixture trajectory together with its two component trajectories. The 2-D plots were derived from a principal component analysis with rotations calculated to maximize the distance of mixture components during the first response peak (Fig. 6b). In some mixtures, one component clearly dominated the mixture response pattern (first two columns in Fig. 6b, where the green trajectory (mix) closely follows either the yellow or the blue trajectory (one component) while in others the mixture was intermediate to the component responses (column 3). In others again we observed a “flipping” behavior with the mixture trajectory initially being intermediate and then flipping to one component (column 4), comparable to the individual mixture response becoming biphasic in Fig. 6a. Interestingly, in these cases, the component of the trajectory that corresponded to one of the components reflected the phase after stimulus offset. In other words, the data showed that in some odor mixtures, one odor was dominant (e.g., when mixing ETBE [ethyl butyrate] and PELM [2-phenylethanol], the component ETBE was dominant, suggesting elemental coding of one element, and suppression of the other), while in other mixtures the trajectory indicated a new, configural odor percept (e.g., when mixing ESHE [ethyl (*S*)-(+)-3-hydroxybutyrate] and ETAS [acetic acid]). Whether the “flipping” behavior might indicate that the animal may have access to both elemental odor percepts would be an intriguing hypothesis deriving from these observations.

## Discussion

We measured OSN responses to mono-molecular odorants and binary mixtures. We found that temporal response dynamics were complex, including phasic and tonic elements, and that they were odorant–OSN combination specific and thus carried information about odorant identity, sufficient to enhance the performance of a classifying algorithm. We also showed that odorant mixtures led to response dynamics with both elemental and configural signatures. Diversity in temporal response dynamics of *Drosophila* OSNs, including biphasic responses, has been observed in previous studies of electrophysiological responses in OSNs but to our knowledge has never been studied comprehensively across many odorants and OSNs (de Bruyne et al. 1999, 2001; Hallem et al. 2004; Kreher et al. 2005; Nagel and Wilson 2011; Grillet et al. 2016).

### Potential origin of complex response dynamics

Several mechanisms can potentially contribute to the odorant–OSN combination-specific response dynamics, and the results that we measured may be a combination of all. (1) Even before hitting a biological surface, odorants delivered by our olfactometer have different physical properties of adsorption to the technical surfaces used, leading to slightly different temporal patterns (Figs. 2b, S2b; Martelli et al. 2013): some substances are more “sticky” than others. These properties will also occur in a natural environment, leading to odors that linger for longer, and others that do not. In a natural environment, another factor will add to this: differences in volatility lead to different diffusion rates of substances, which alters their temporal profile at the OSN. And finally, differences in adsorption will also occur on the cuticular surface of the animal, which is an effect that we did not measure in this study. (2) In addition, sensillar events influence the temporal processes that transport odorant molecules from the surface to the OSN dendrites (Syed et al. 2006; Chertemps et al. 2012; Kaissling 2013; Rospars 2013; Larter et al. 2016), including affinity to odorant binding proteins, and solubility to the sensillar lymph. The importance of the sensillar lymph is evident from studies that show that ectopical expression of receptors in other sensilla only works within particular sensilla types, showing that each receptor needs a specific sensillar complement (Ronderos et al. 2014). (3) receptor affinity and saturation curves are likely to be the main factors affecting odorant-response time courses. These have been studied extensively in ligand-receptor studies of metabotropic receptors (Kenakin 2017). The direct interaction of the odorant molecule with the receptor protein is most likely the principal factor determining whether the response is phasic or has tonic elements: in pharmacological characterizations of receptor–ligand interactions, time constants for binding and for release can have different values (Tummino and Copeland 2008). (4) negative and positive responses (inhibitions or excitations) arise from competitive binding of ligand and receptor. In particular, a ligand that displaces a tonically present alternative ligand, or that allosterically competes with another ligand, will lead to different temporal profiles. Several other molecular mechanisms for inhibitory responses are possible (e.g., inhibitory transduction cascades, Michel and Ache 1992, “leaky” receptors that are closed, Costa and Herz 1989, etc.). (5) The transduction cascade might add to the temporal properties. In the present study, we do not expect the transduction cascade to differ between seven receptors used, since they all share the same Orco co-receptor, and can all be expressed ectopically in Or22a neurons, without apparent loss in function (Dobritsa et al. 2003; Kreher et al. 2008; Dweck et al. 2015; Lebreton et al. 2017). Whether the 8th receptor, Or56a, would function in the Or22a empty-neuron is not known since its best ligand, geosmin (Stensmyr et al. 2012), has not been tested in that system yet. However, in the full olfactory system of *Drosophila*, we anticipate the diversity to be much larger, given that different ORs might have different transduction pathways, e.g., different G proteins involved in either transduction itself or in regulating transduction (Wicher et al. 2008; Yao and Carlson 2010; Ignatious Raja et al. 2014), and that IRs probably have a different transduction cascade altogether (Benton et al. 2009). Together, these considerations suggest that the role of temporal diversity might even be underestimated in our sample of eight OSN classes and 99 odorants.

The major factor affecting temporal response properties is adaptation. Sensory neurons with fast adaptation show phasic responses, slow adaptation leads to tonic responses. In our samples, we found all sorts of intermediate cases: very phasic responses (e.g., Or56a to PRBL, Fig. 2), and very slow, long lasting responses (e.g., Or10a to 2EBM). We included a direct test for adaptation by giving a double-pulse stimulus: the second pulse, given 3 s after the first pulse, was a test of the response in a adapted state. The fact that we found a great variety of different behaviors to the second pulse, dependent both on the OSN class, and on the odorant, suggests that adaptation resides to a large degree in a mechanism that involves the odorant molecule itself, most likely receptor–ligand interaction, though we cannot exclude a contribution from the sensillar lymph. We note that the sensory neuron with the most biphasic (i.e. complex) responses was Or56a. These cells co-express a further receptor, Or33a (Fishilevich and Vosshall 2005), for which no functional role has been reported yet. A contribution to temporal complexity is an intriguing hypothesis to be tested.

The diverse temporal response dynamics we observed were stable across a range of concentrations. This is interesting as the overall activation pattern across OSNs changes with odorant concentration (Sachse and Galizia 2003; Silbering et al. 2008; Strauch and Galizia 2012). Thus, response dynamics could facilitate concentration-independent coding of odorant identity.

This study has several limitations that need to be taken into account when interpreting the data. Most importantly, any study in olfactory coding that looks at the “entire” olfactory landscape is limited, since that landscape is vast. Specifically, eight OSNs are many, but still only 16% of all fly OSN classes, 99 odorants might sound a lot, but still they are a minute fraction of olfactory space, and binary odorants are but a glimpse on the complexity of odorant mixture. Similarly, probing adaptation with a single, second pulse after 2.75 s does not allow to study the temporal development of adaptation, nor the effects of cross adaptation. Furthermore, our experimental design does not allow to analyze the effect of temporally complex plume structures onto olfactory coding. However, despite all of these limitations, this study already offers an astounding view on the diversity in temporal responses, and suggests that animals might use time to enhance their sensory capacity.

### Impact on olfactory coding

We show here that time increases the coding capacity of the olfactory system. Specifically, adding information about the temporal development of an odor response across OSNs increases the capacity to identify the stimulus, from 33 to 45% (Fig. 5). This increased capacity comes at a cost, the animal needs to wait until the brain can evaluate the temporally evolving pattern. Since the initial, phasic odor-response pattern already contains a large amount of information, this raises an important question: do animals evaluate odor information using the initial response peak (high-speed, low-accuracy), or do they wait for the pattern to evolve (low-speed, high-accuracy)? In honeybees and rodents, time-to-decision was measured to be constant irrespective of odor-choice difficulty (Uchida and Mainen 2003; Ditzen et al. 2003), though other experiments found that there is a speed-accuracy trade off and odor classification becomes more reliable when more time is available (Abraham et al. 2004). This result suggests that an animal can decide, and taking time into consideration represents a trade-off: accuracy against speed (Heitz 2014). We are not aware of experiments in Drosophila that have analyzed this fact. We do not expect the temporal constants to be equal across species and environmental situations, however. In different fly species, visual transduction differs in speed, with *D. melanogaster* being on the slow and energetically cheap side (Niven et al. 2007). Whether an animal uses fast or slow odor evaluation may depend on the situation: insects can extract mixture-component information from stimuli where the components are separated by temporal differences as short as 6 ms (Szyszka et al. 2014). In such a situation, fast coding is necessary. On the other hand, Drosophila will find an odor source (say, a glass of wine), in a room without major turbulences, in a meandering flight, a situation where slow coding is sufficient. The brain may add to the temporal complexity that we have measured here across sensory neurons. For example, in zebra fish, combinatorial patterns in the olfactory bulb evolve and increase their information content during the first ${\sim }$800 ms of the response (Friedrich and Laurent 2001).

The response strength that a mixture of odorants would elicit from an OSN is not easily predictable from the components’ responses due to different kinds of mixture interactions (Silbering and Galizia 2007; Rospars et al. 2008; Hillier and Vickers 2011; Münch et al. 2013). Here, we show that not only response strength differs for different odorant combinations but also new response dynamics might arise from mixing two odorants. Some mixture responses in our data were first dominated by the excitatory component and later by the inhibitory which might be a consequence of sharpening of the excitatory response by the inhibitory component (Su et al. 2011). Potentially, such a response could convey information on both components of a mixture, allowing for double elemental mixture analysis. Behavioral data found that discriminability of components within a mixture depends on the identity of the components, since some studies show elemental mixture coding, others configural mixture coding. The data reported here indicate that this distinction arises already at the level of the olfactory sensory neurons, suggesting that—at least for some cases—the brain might not be able to switch from elemental to configural analysis.

## Electronic supplementary material

Below is the link to the electronic supplementary material.

Supplementary material 1 (PDF 2440 kb)

## Abbreviations

$\Delta F/F$: Relative fluorescence change
OSN: Olfactory sensory neuron
PCA: Principle component analysis
PID: Photoionization detector
