# Supplementary material for: Take time: odor coding capacity across sensory neurons increases over time in Drosophila
Source: J Comp Physiol A Neuroethol Sens Neural Behav Physiol. 2017 Aug 29;203(12):959–72. doi: 10.1007/s00359-017-1209-1 (PMC5696509; doi:10.1007/s00359-017-1209-1)
Supplement: Supplementary file 1 — Supplementary material 1 (PDF 2440 kb) [file 359_2017_1209_MOESM1_ESM.pdf]

Take time: odor coding capacity across sensory neurons increases over time in *Drosophila*

Journal of Comparative Physiology A

Supplemental Material

Daniel Münch[1,2] and C. Giovanni Galizia[1]

[1]Neurobiology, University of Konstanz, 78457 Konstanz, Germany
[2]daniel.muench@uni-konstanz.de

August 11, 2017

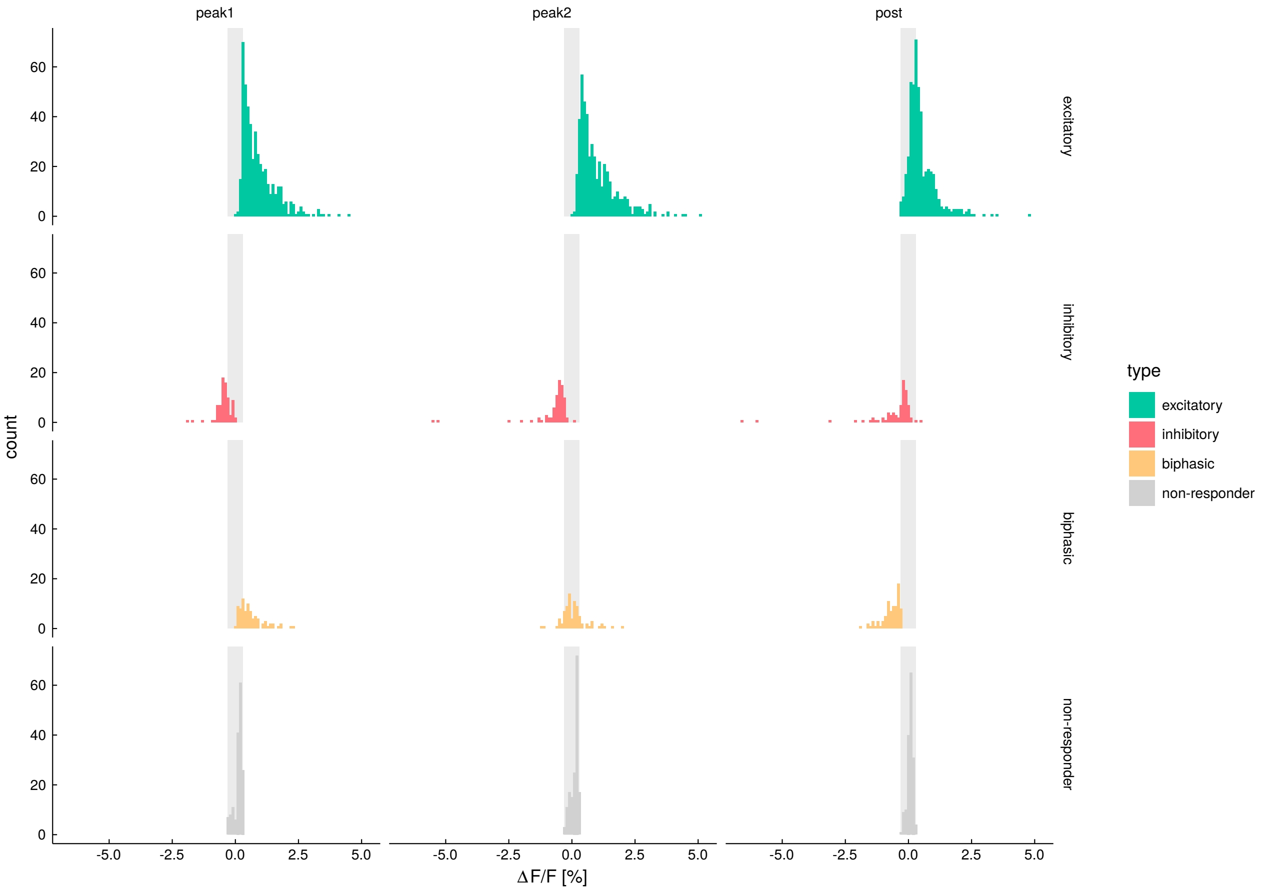

Figure S1 Related to Figure 1. Histogram of response strength distributions. Colors indicate the response type classification, *gray* shades highlight the $|0.3\%|\Delta F/F$ ($\pm 2.5 \times SD$ before stimulus onset) threshold that was used to classify non-responders (see Material & Methods)

Table S1 List of odorants used in the different experiments

CODE	Name	InChIKey	data set
2EBM	ethyl benzoate	MTZQAGJQAFMTAQ-UHFFFAOYSA-N	99 odorants
2EPM	2-ethylphenol	IXQGCWUGDFDQMF-UHFFFAOYSA-N	99 odorants
2PPM	2-propylphenol	LCHYEKKJCUJAKN-UHFFFAOYSA-N	99 odorants
3HXN	3-hexanone	PFCHFHIRKBAQGU-UHFFFAOYSA-N	99 odorants
4MPM	4-methylphenol	IWDCLRJOBJJRNH-UHFFFAOYSA-N	99 odorants
AIOT	α-ionone	UZFLPKAIBPNNCA-BQYQJAHWSA-N	99 odorants
AIR	room air		99 odorants
BACE	butyl acetate	DKPFZGUDAPQIHT-UHFFFAOYSA-N	99 odorants
BBTL	β-butyrolactone	GSCLMSFRWBPUSK-UHFFFAOYSA-N	99 odorants
BDOL	2,3-butanediol	OWBTYPJTUOEWEK-UHFFFAOYSA-N	99 odorants
BEAM	benzaldehyde	HUMNYLRZRPPJDN-UHFFFAOYSA-N	99 odorants
BEDN	2,3-butanedione	QSJXEFYPDANLFS-UHFFFAOYSA-N	99 odorants
BIOT	β-ionone	PSQYTAPXSHCGMF-BQYQJAHWSA-N	99 odorants
BIST	α-bisabolol	RGZSQWQPBWRIAQ-CABCVRRESA-N	99 odorants
BNIM	benzonitrile	JFDZBHWFFUWGJE-UHFFFAOYSA-N	99 odorants
BOLM	benzyl alcohol	WVDDGKGOMKODPV-UHFFFAOYSA-N	99 odorants
BUTA	butanal	ZTQSAGDEMFDKMZ-UHFFFAOYSA-N	99 odorants
BUTN	2-butanone	ZWEHNKRNPOVVGH-UHFFFAOYSA-N	99 odorants
CAPT	(-)-trans-caryophyllene	NPNUFJAVOOONJE-GFUGXAQUSA-N	99 odorants
CART	(R)-(-)-carvone	ULDHMXUKGWMISQ-SECBINFHSA-N	99 odorants
CAST	(S)-(+)-carvone	ULDHMXUKGWMISQ-VIFPVBQESA-N	99 odorants
CIAT	(S)-(-)-citronellal	NEHNMFOYXAPHSD-JTQLQIEISA-N	99 odorants
CINT	1,8-cineole	WEEGYLXZBRQIMU-WAAGHKOSSA-N	99 odorants
CITT	citral	WTEVQBCEXWBHNA-JXMROGBWSA-N	99 odorants
CYHN	cyclohexanone	JHIVVAPYMSGYDF-UHFFFAOYSA-N	99 odorants
DECA	decanal	KSMVZQYAVGTKIV-UHFFFAOYSA-N	99 odorants
DECL	decanol	MWKFXSUHUHTGQN-UHFFFAOYSA-N	99 odorants
DMBM	4-allyl-1,2-dimethoxybenzene	ZYEMGPIYFIJGTP-UHFFFAOYSA-N	99 odorants
E3HE	ethyl 3-hydroxyhexanoate	LYRIITRHDCNUHV-UHFFFAOYSA-N	99 odorants
EHAE	E2-hexenyl acetate	HRHOWZHRCRZVCU-WAYWQWQTSA-N	99 odorants
EIVE	ethyl isovalerate	PPXUHEORWJQRHJ-UHFFFAOYSA-N	99 odorants
EM2E	ethyl 2-methyl-2e-butenoate	OAPHLAAOJMTMLY-GQCTYLIASA-N	99 odorants
EMBE	ethyl 2-methylbutyrate	HCRBXQFHJMCTLF-UHFFFAOYSA-N	99 odorants
ERHE	ethyl (R)-(-)-3-hydroxybutyrate	OMSUIQOIVADKIM-RXMQYKEDSA-N	99 odorants
ESHE	ethyl (S)-(+)-3-hydroxybutyrate	OMSUIQOIVADKIM-YFKPBYRVSA-N	99 odorants
ETAS	acetic acid	QTBSBXVTEAMEQO-UHFFFAOYSA-N	99 odorants
EUGM	eugenol	RRAFCDWBNXTKKO-UHFFFAOYSA-N	99 odorants
FART	E,E-farnesol	CRDAMVZIKSXKFV-YFVJMOTDSA-N	99 odorants
FENT	(1R)-(-)-fenchone	LHXDLQBQYFFVNW-OIBJUYFYSA-N	99 odorants
FURL	furfural	HYBBIBNJHNGZAN-UHFFFAOYSA-N	99 odorants
GERT	geraniol	GLZPCOQZEFWAFX-JXMROGBWSA-N	99 odorants
GVAL	γ-valerolactone	GAEKPEKOJKCEMS-UHFFFAOYSA-N	99 odorants
HEPA	heptanal	FXHGMKSSBGDXIY-UHFFFAOYSA-N	99 odorants
HEPK	heptane	IMNFDUFMRHMDMM-UHFFFAOYSA-N	99 odorants
HEPN	2-heptanone	CATSNJVOTSVZJV-UHFFFAOYSA-N	99 odorants
HEPS	heptanoic acid	MNWFXJYAOYHMED-UHFFFAOYSA-N	99 odorants
HEXA	hexanal	JARKCYVAAOWBJS-UHFFFAOYSA-N	99 odorants
HEXN	2-hexanone	QQZOPKMRPOGIEB-UHFFFAOYSA-N	99 odorants
HEXS	hexanoic acid	FUZZWVXGSFPDMH-UHFFFAOYSA-N	99 odorants
HP2L	2-heptanol	CETWDUZRCINIHU-UHFFFAOYSA-N	99 odorants
HPAE	heptyl acetate	ZCZSIDMEHXZRLG-UHFFFAOYSA-N	99 odorants
HX2A	E2-hexenal	MBDOYVRWFFCFHM-SNAWJCMRSA-N	99 odorants
HX2L	2-hexanol	QNVRIHYSUZMSGM-UHFFFAOYSA-N	99 odorants
HX3L	1-hexen-3-ol	BVOSSZSHBZQJOI-UHFFFAOYSA-N	99 odorants
HXAE	hexyl acetate	AOGQPLXWSUTHQB-UHFFFAOYSA-N	99 odorants

Table S1 *continued*

CODE	Name	InChIKey	data set
HXBE	hexyl butyrate	XAPCMTMQBXLDBB-UHFFFAOYSA-N	99 odorants
HXME	hexyl methanoate	OUGPMNMLWKSBRI-UHFFFAOYSA-N	99 odorants
IATE	isoamyl tiglate	ZARFDQHJMNVNLE-WEVVVXLNSA-N	99 odorants
IEUM	iso-eugenol	BJIOGJUNALELMI-ONEGZZNKSA-N	99 odorants
IPBE	isopropyl butyrate	FFOPEPMHKILNIT-UHFFFAOYSA-N	99 odorants
IPTE	isopropyl tiglate	VUPBIVVRPJDWNW-FNORWQNLSA-N	99 odorants
LIMT	(R)-(+)-limonene	XMGQYMWWDOXHJM-JTQLQIEISA-N	99 odorants
LINT	linalool	CDOSHBSSFJOMGT-UHFFFAOYSA-N	99 odorants
M3HE	methyl 3-hydroxy hexanoate	ACCRBMDJCPPJDX-UHFFFAOYSA-N	99 odorants
MBAE	2-methyl butyl acetate	XHIUFYZDQBSEMF-UHFFFAOYSA-N	99 odorants
MBAM	4-methoxybenzaldehyde	ZRSNZINYAWTAHE-UHFFFAOYSA-N	99 odorants
MBUL	3-methyl-butanol	PHTQWCKDNZKARW-UHFFFAOYSA-N	99 odorants
MCHL	4-methylcyclohexanol	MQWCXKGKQLNYQG-UHFFFAOYSA-N	99 odorants
MEBM	4-methoxybenzene	RDOXTESZEPMUJZ-UHFFFAOYSA-N	99 odorants
MJSM	methyl jasmonate	GEWDNTWNSAZUDX-SNAWJCMRSA-N	99 odorants
MOL	mineral oil	solvent	99 odorants
MPRS	isobutyric acid	KQNPFQTWMSNSAP-UHFFFAOYSA-N	99 odorants
MSAM	methyl salicylate	OSWPMRLSEDHDFF-UHFFFAOYSA-N	99 odorants
MTIE	methyl tiglate	YYJWBYNQJLBIGS-PLNGDYQASA-N	99 odorants
MYRT	(1R)-(-)-myrtenal	KMRMUZKLFIEVAO-UHFFFAOYSA-N	99 odorants
NONA	nonanal	GYHFUZHODSMOHU-UHFFFAOYSA-N	99 odorants
NONK	nonane	BKIMMITUMNQMOS-UHFFFAOYSA-N	99 odorants
NONN	nonanone	VKCYHJWLYTUGCC-UHFFFAOYSA-N	99 odorants
NONS	nonanoic acid	FBUKVWPVBMHYJY-UHFFFAOYSA-N	99 odorants
OC3L	3-octanol	NMRPBPVERJPACX-UHFFFAOYSA-N	99 odorants
OC4L	4-octanol	WOFPPJOZXUTRAU-UHFFFAOYSA-N	99 odorants
OCTA	octanal	NUJGJRNETVAIRJ-UHFFFAOYSA-N	99 odorants
OCTK	octane	TVMXDCGIABBOFY-UHFFFAOYSA-N	99 odorants
OCTN	2-octanone	ZPVFWPFBNIEHGJ-UHFFFAOYSA-N	99 odorants
OCTS	octanoic acid	WWZKQHOCKIZLMA-UHFFFAOYSA-N	99 odorants
P2ON	3-penten-2-one	LABTWGUMFABVFG-ARJAWSKDSA-N	99 odorants
PANM	4-propenyl anisole	RUVINXPYWBROJD-ONEGZZNKSA-N	99 odorants
PENA	pentanal	HGBOYTHUEUWSSQ-UHFFFAOYSA-N	99 odorants
PENS	pentanoic acid	NQPDZGIKBAWPEJ-UHFFFAOYSA-N	99 odorants
PINT	(+)-α-pinene	GRWFGVWFFZKLTI-RKDXNWHRSA-N	99 odorants
PRBL	γ-propyl-γ-butyrolactone	VLSVVMPLPMNWBH-UHFFFAOYSA-N	99 odorants
PROA	propanal	NBBJYMSMWIIQGU-UHFFFAOYSA-N	99 odorants
PRON	acetone	CSCPPACGZOOCGX-UHFFFAOYSA-N	99 odorants
PROS	propanoic acid	XBDQKXXYIPTUBI-UHFFFAOYSA-N	99 odorants
PULT	(R)-(+)-pulegon	NZGWDASTMWDZIW-MRVPVSSYSA-N	99 odorants
TERT	α-terpineol	WUOACPNHFRMFPN-SECBINFHSA-N	99 odorants
THUT	(-)-α-thujone	USMNOWBWPHYOEA-MRTMQBJTSA-N	99 odorants
Z3HL	Z3-hexenol	UFLHIIWVXFIJGU-ARJAWSKDSA-N	99 odorants
ZHAE	Z3-hexenyl acetate	NPFVOOAXDOBMCE-PLNGDYQASA-N	99 odorants
ESHE	ethyl (S)-(+)-3-hydroxybutyrate	OMSUIQOIVADKIM-YFKPBYRVSA-N	mix set 1
ETAS	acetic acid	QTBSBXVTEAMEQO-UHFFFAOYSA-N	mix set 1
GEST	geranyl acetate	HIGQPQRQIQDZMP-DHZHZOJOSA-N	mix set 1
PROA	propanal	NBBJYMSMWIIQGU-UHFFFAOYSA-N	mix set 1
Z3HL	Z3-hexenol	UFLHIIWVXFIJGU-ARJAWSKDSA-N	mix set 1
ETBE	ethyl butyrate	OBNCKNCVKJNDBV-UHFFFAOYSA-N	mix set 2
ISOE	isopentyl acetate	MLFHJEHSLIIPHL-UHFFFAOYSA-N	mix set 2
FURL	furfural	HYBBIBNJHNGZAN-UHFFFAOYSA-N	mix set 2
PE2L	2-pentanol	JYVLIDXNZAXMDK-UHFFFAOYSA-N	mix set 2
PELM	phenethyl alcohol	WRMNZCZEMHIOCP-UHFFFAOYSA-N	mix set 2

Table S2 Related to Fig. 5. List of odorants sorted according to overall strength, see Material & Methods for details regarding the calculation of "overall strength"

rank	set 1	rank	set 2	rank	set 3	rank	set 4
1	BEDN	26	HXME	51	HX2A	75	DMBM
2	ESHE	27	M3HE	52	THUT	76	HPAE
3	HX2L	28	OC4L	53	E3HE	77	OCTK
4	ETAS	29	BOLM	54	IATE	78	HEXA
5	P2ON	30	CINT	55	MBAE	79	2PPM
6	MCHL	31	HEPN	56	PANM	80	PINT
7	ERHE	32	GVAL	57	OCTN	81	CYHN
8	PROS	33	LINT	58	MBUL	82	LIMT
9	3HXN	34	PULT	59	HEXN	83	HEPK
10	HP2L	35	EMBE	60	GERT	84	NONK
11	2EBM	36	BACE	61	CAPT	85	BUTA
12	Z3HL	37	FURL	62	BUTN	86	IPTE
13	FENT	38	CAST	63	BIOT	87	BIST
14	BEAM	39	BNIM	64	FART	88	HEPA
15	MTIE	40	MBAM	65	PENA	89	DECA
16	PRBL	41	CITT	66	EHAE	90	OCTA
17	OC3L	42	MPRS	67	HXBE	91	IPBE
18	MSAM	43	CIAT	68	HXAE	92	PRON
19	PROA	44	EIVE	69	DECL	93	IEUM
20	HX3L	45	PENS	70	EUGM	94	MOL
21	BDOL	46	CART	71	NONN	95	HEPS
22	BBTL	47	MEBM	72	MJSM	96	HEXS
23	TERT	48	ZHAE	73	MYRT	97	NONS
24	EM2E	49	2EPM	74	NONA	98	AIR
25	AIOT	50	4MPM	75	DMBM	99	OCTS

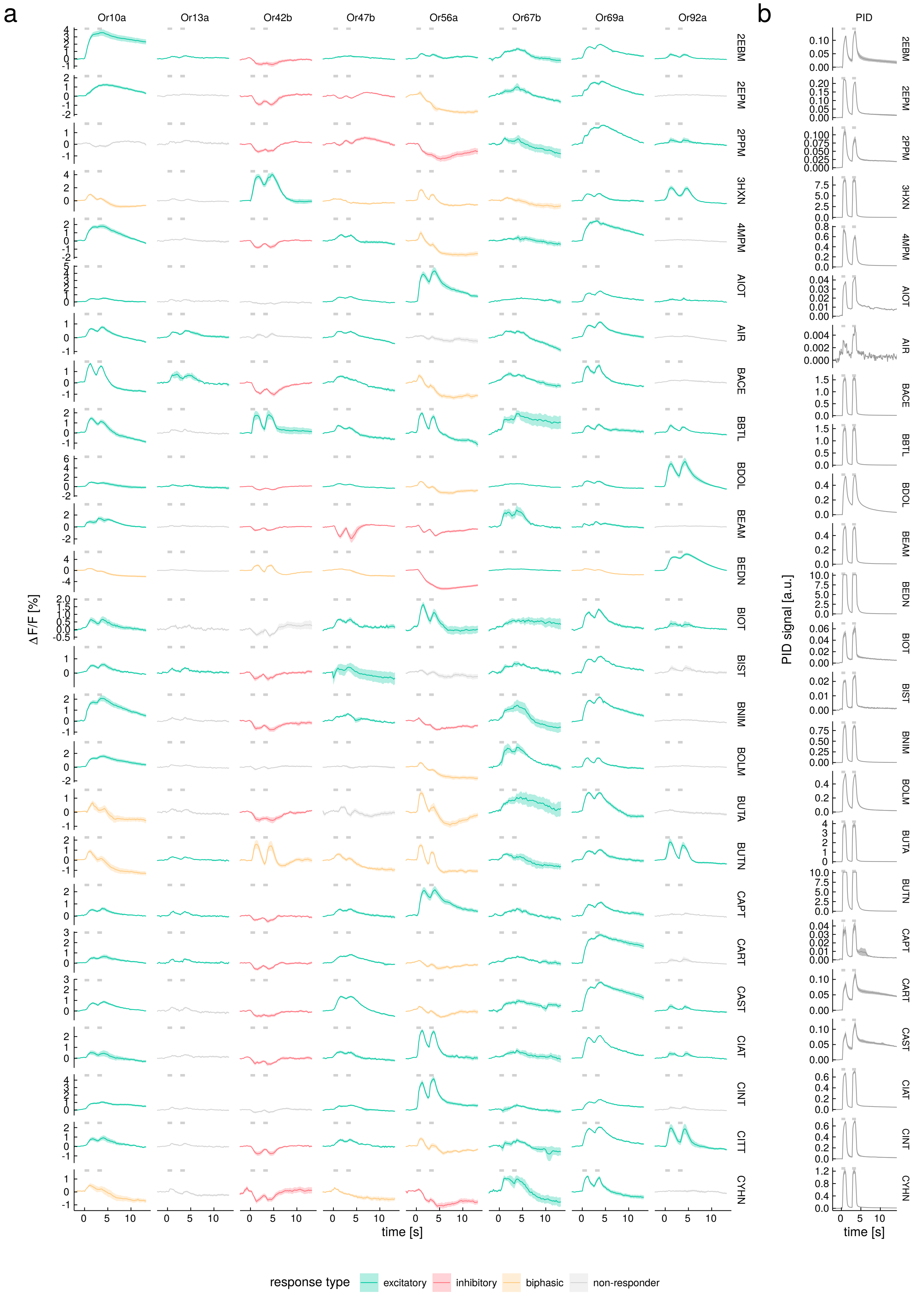

Figure S2 Related to Figure 2. **a** Response traces for all odorant-OSN combintions tested. Traces are given as average of n = 3-28 animals (median = 8), shades indicate SEM. **b** PID recordings of 99 odorants tested. Traces are given as average of n = 1-3 independent measurements (median = 3), shades indicate SEM

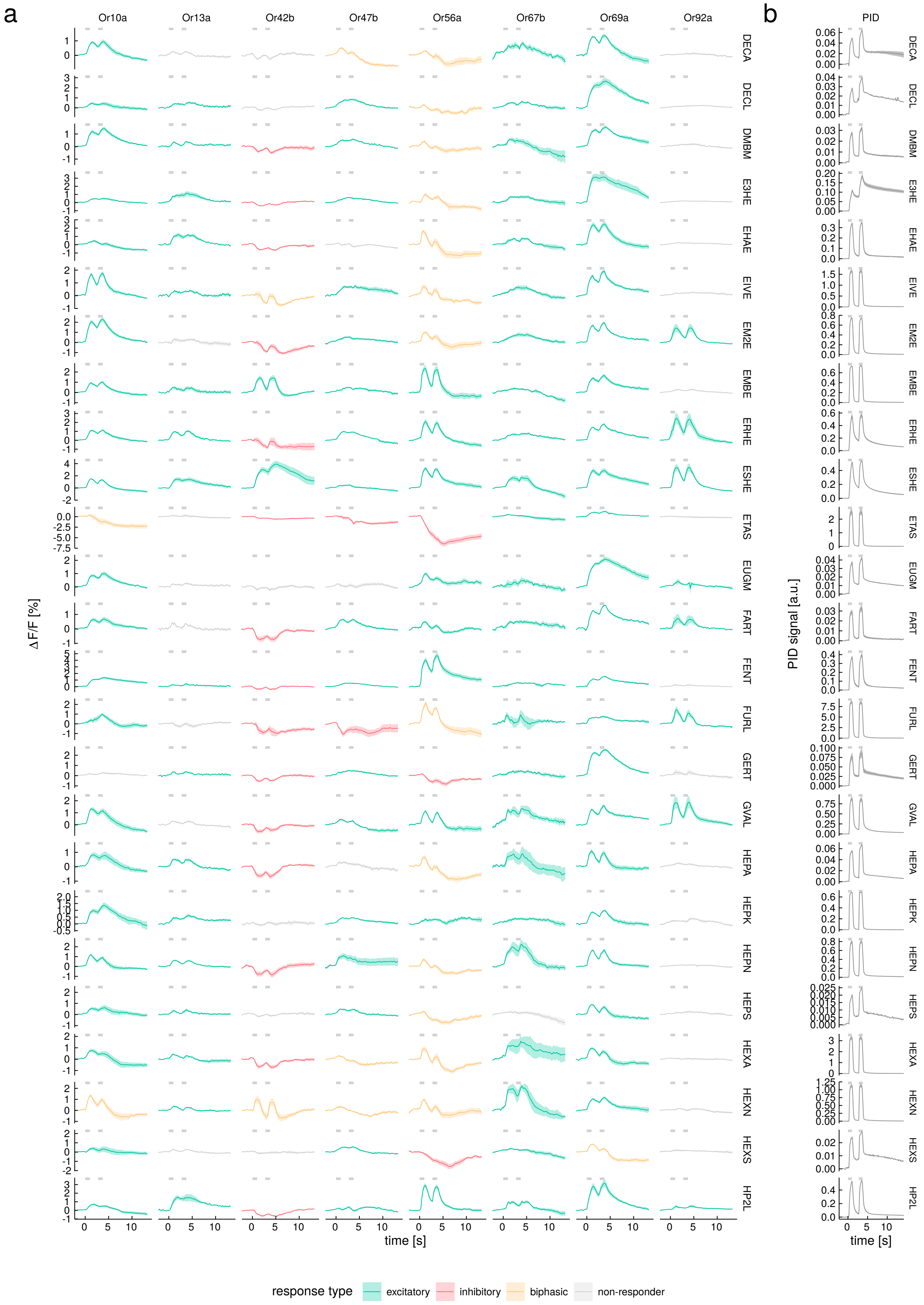

Figure S2 *continued*

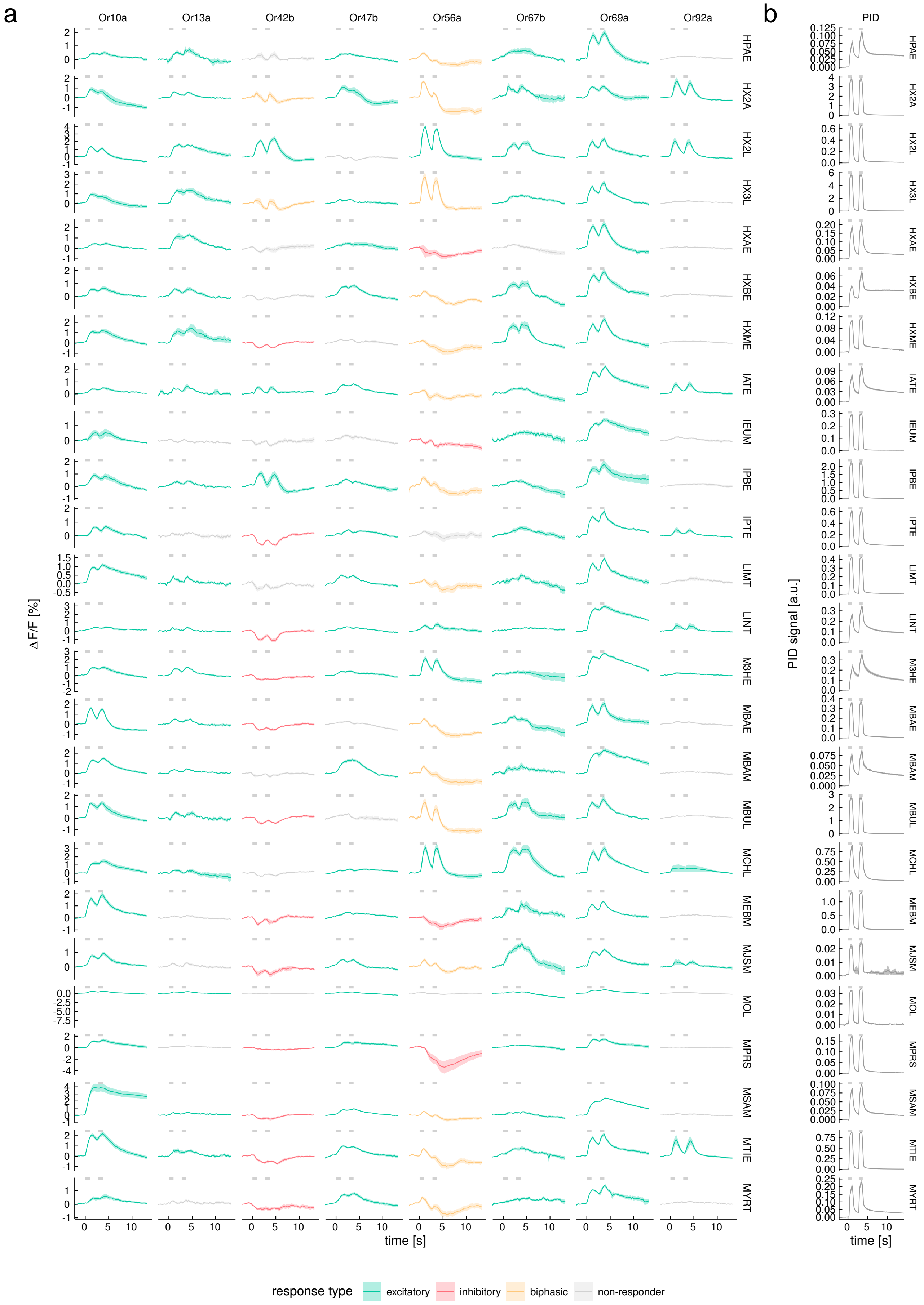

Figure S2 *continued*

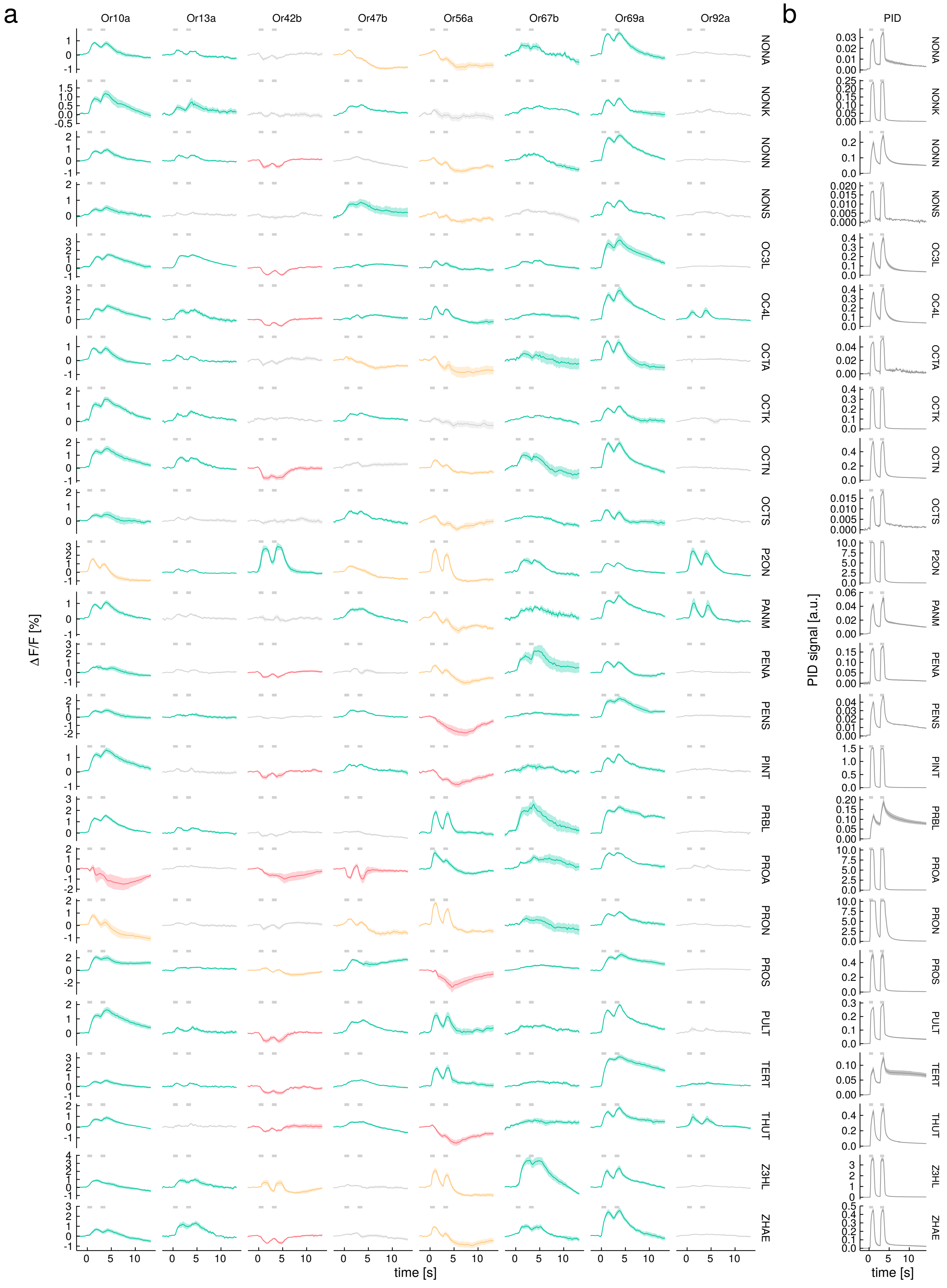

Figure S2 *continued*

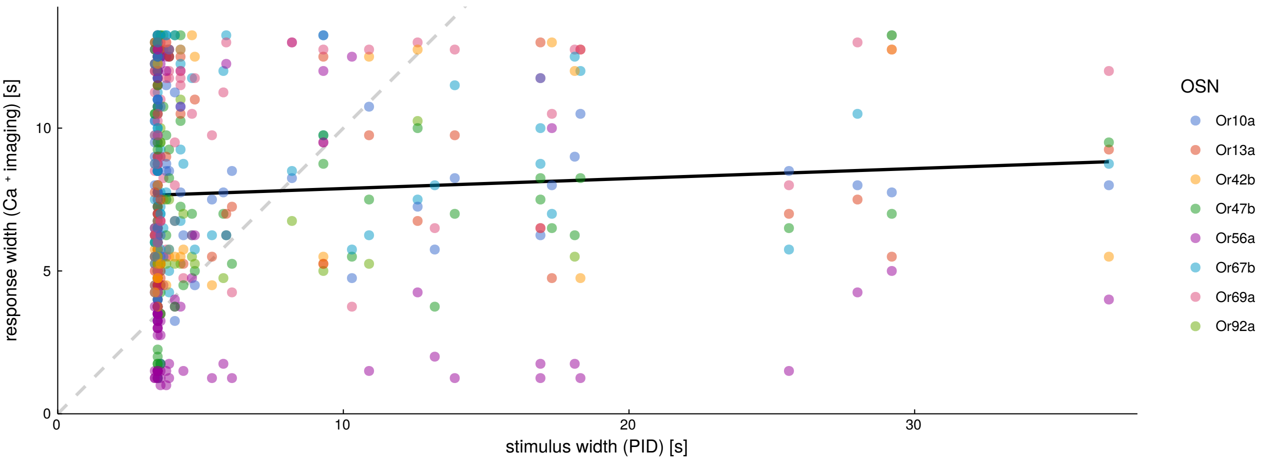

Figure S3 Related to Figure 2. Response width vs stimulus width. Widths of stimuli and responses were not correlated (Pearson's correlation coefficient r = 0.06, p = 0.15). Response width was calculated from average response traces (n = 3-295, median = 8), stimulus duration from mean PID measurements (n = 1-3, median = 3). Widths were calculated as the time during which the calcium response or PID measurement was larger than 30 % peak response ($response > 0.3 * |response_{peak}|$). Only responses categorized as "responders" were included in this analysis. The *gray dashed* line indicates unity, the *black* line shows a linear fit. Colors indicate the OSN that was recorded

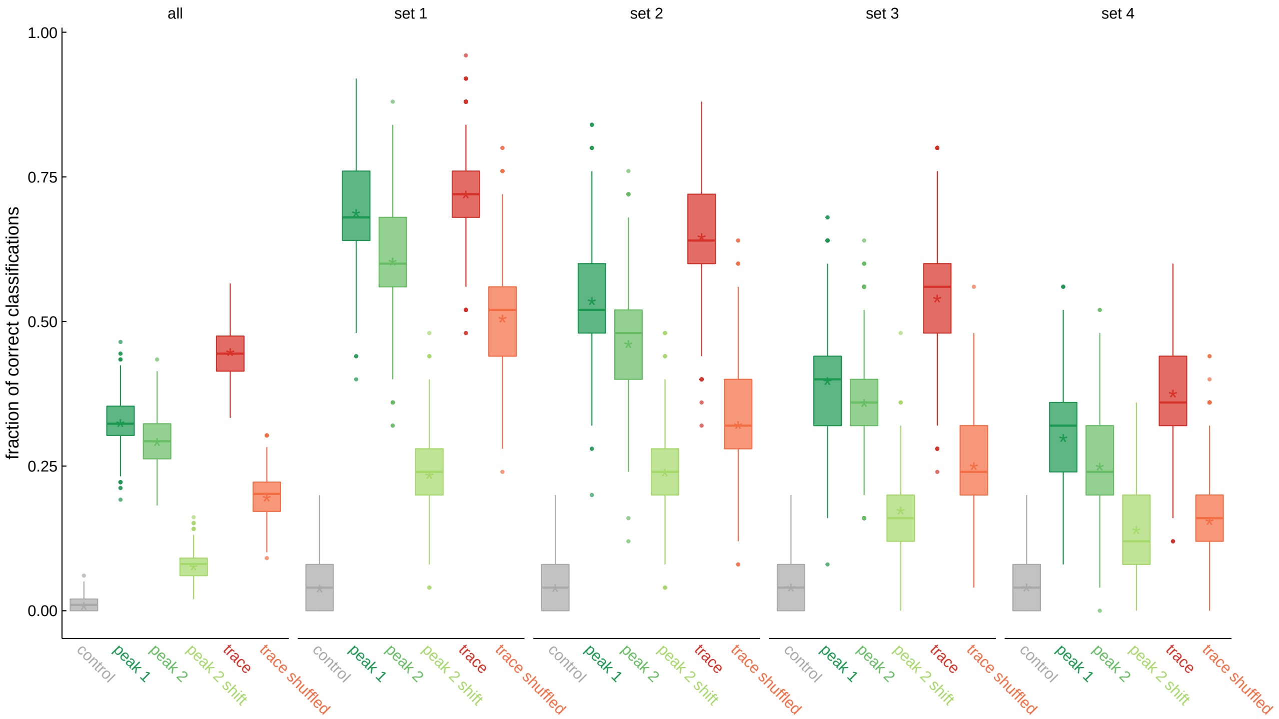

Figure S4 Related to Figure 5. Boxplot of the classifier performance at different time-points. *control* classification with shuffled odor-labels, *peak 1* & *peak 2* five time-points around the 1st and 2nd response peak, *peak 2 shift* classification run on peak 2 but with the activity right prior to peak 2 shifted to 0, *trace* five time-points spread across the recording, *trace shuffled* classification run on the *trace* frames but with scrambled time information. *all* comprises all odorants, *set 1* to *set 4* are subsets derived by ordering odorants according to the overall strength they elicited across the eight sensory neurons (quantified as mean absolute peak response across OSNs), with *set 1* containing the 25 strongest odorants and *set 4* the 25 weakest odorants (see Tab. S2 for a list of all odorants). All differences between classifications at different time points were significant (Kruskal-Wallis rank sum test with a Bonferroni corrected Dunn's post-hoc test, $p < 0.05$). *Boxplots* indicate median, lower and upper quartile, *whiskers* extend to the lowest and highest values that lie within 1.5 times the inter-quartile range from the box, data beyond the whiskers are treated as outliers and indicated as *points*, a*sterisks* indicate the mean

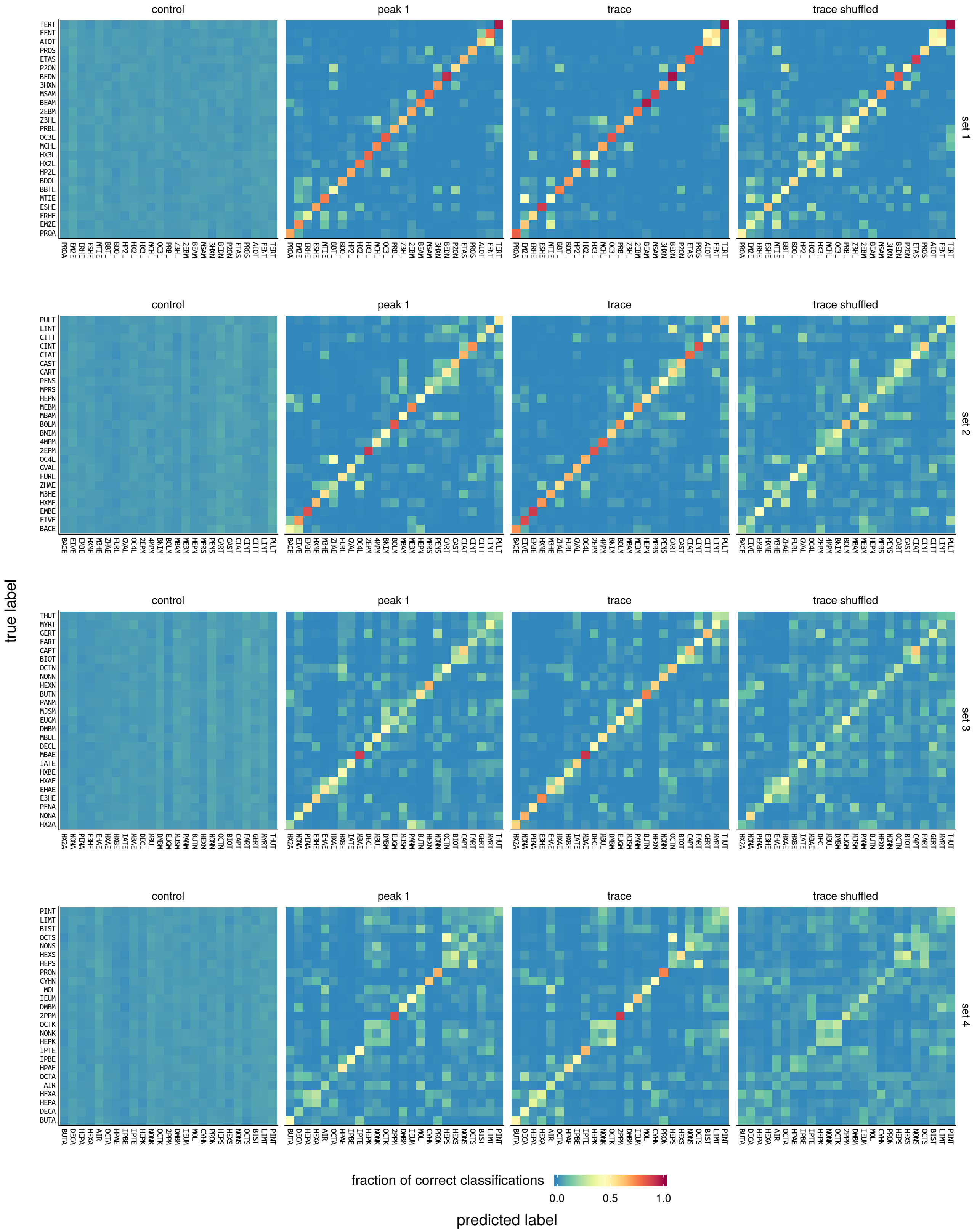

Figure S5 Related to Figure 5. Correct classifications and classification errors in the sets of the strongest and the weakest odorants at the different time-points, visualized as confusion matrices. The values along the diagonal represent classification reliability. See Tab. S1 for a complete list of odorant names & abbreviations.

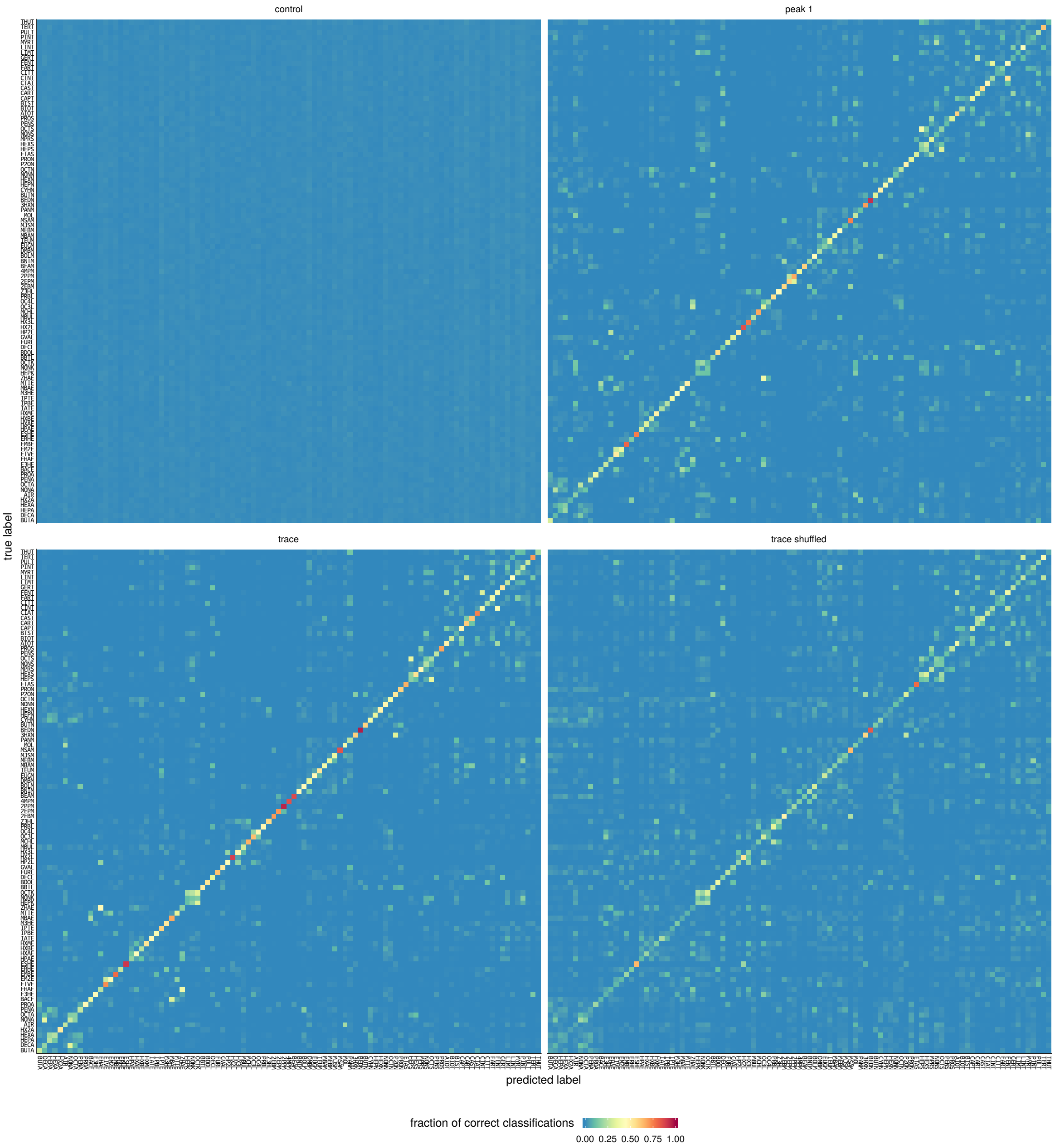

Figure S6 Related to Figure 5. Correct classifications and classification errors in the sets of the strongest and the weakest odorants at the different time-points, visualized as confusion matrices. The values along the diagonal represent classification reliability. See Tab. S1 for a complete list of odorant names & abbreviations

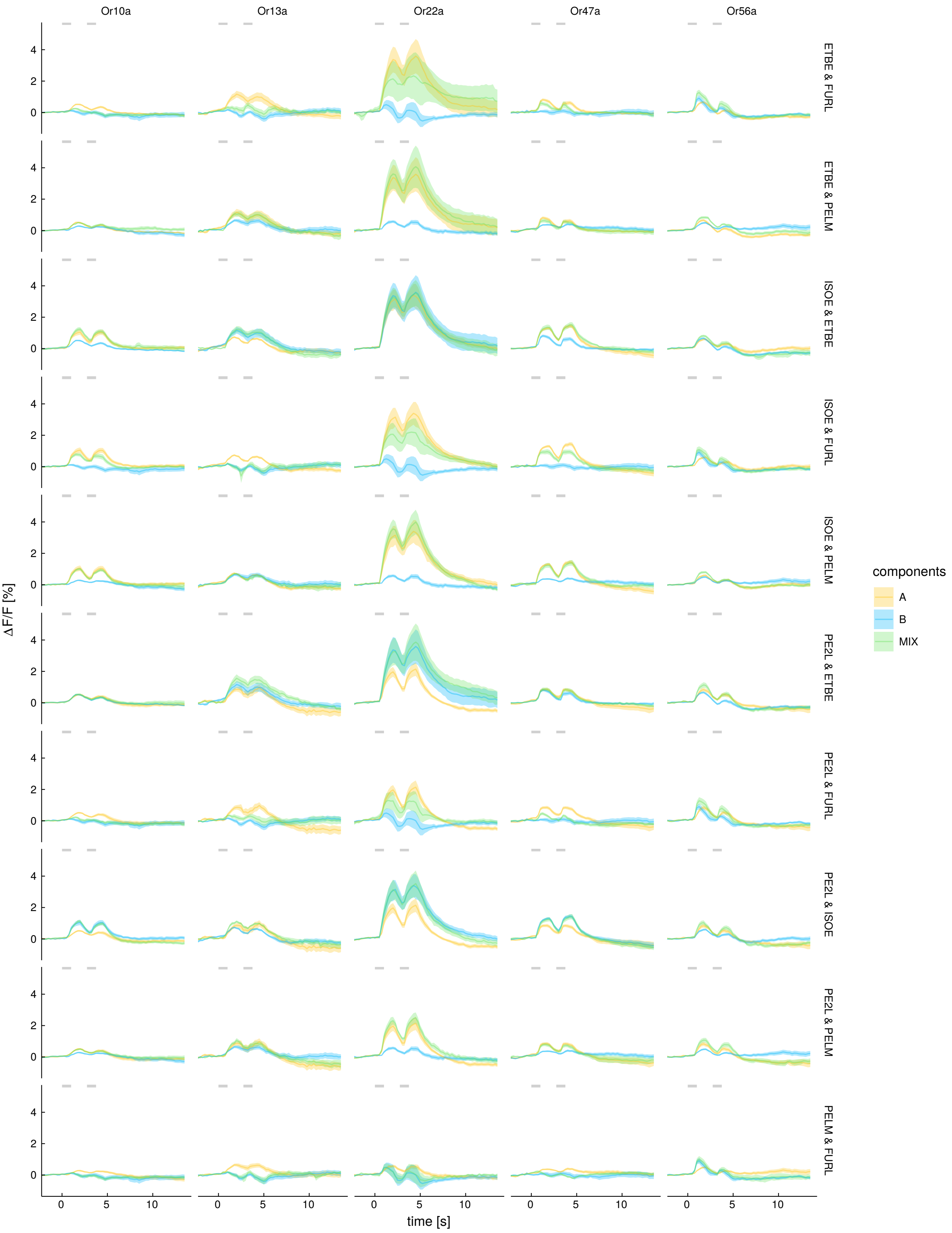

Figure S7 Related to Figure 6. Response traces elicited from binary mixtures of odorants. The mixture trace is shown in *green*, the components are shown in *yellow* and *blue*, *gray* segments indicate the stimulation times. Concentration of the components was the same when tested alone or in the mixture (1×10^{-3} *vol*/*vol* dilution). n = 5-18

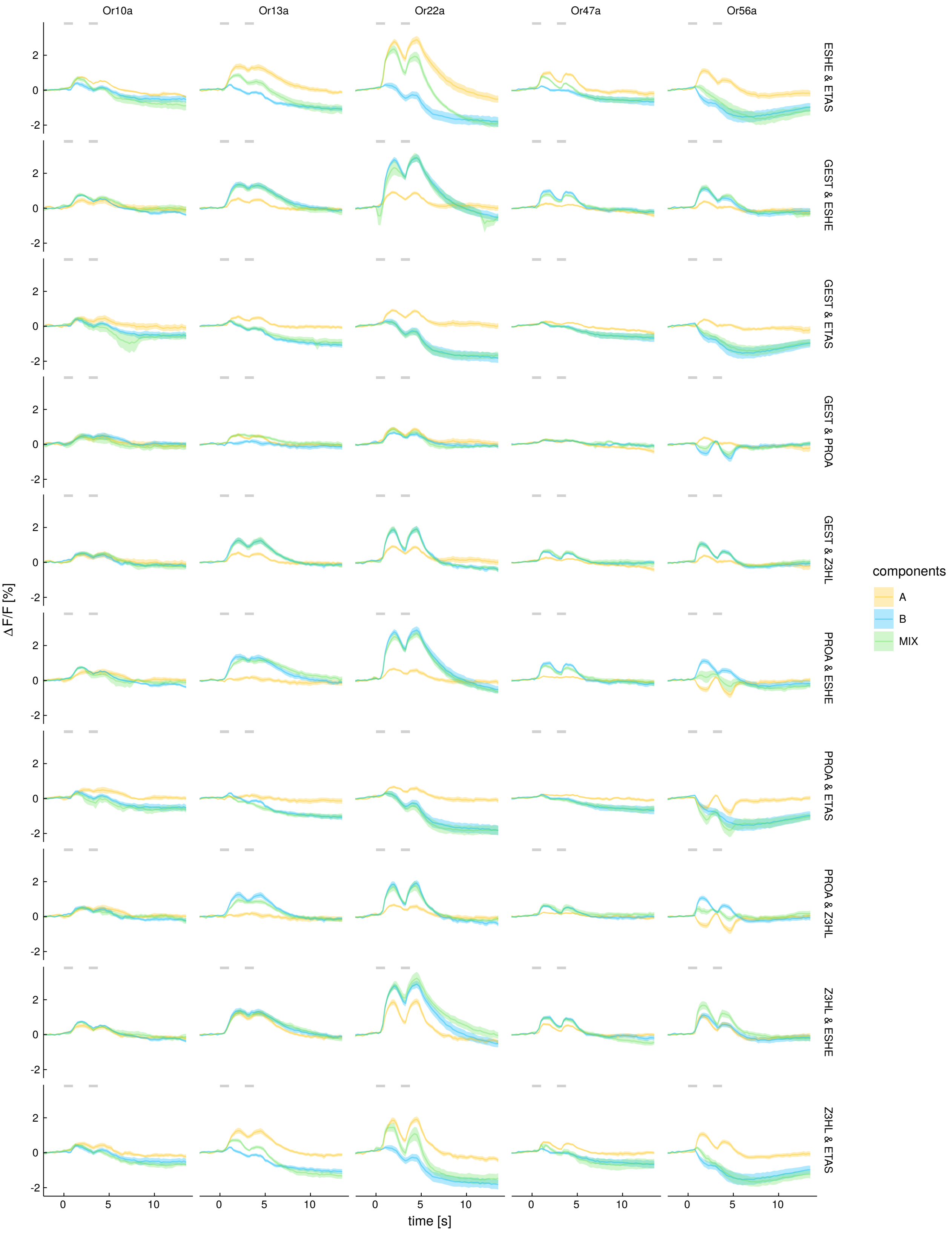

Figure S7 *continued*

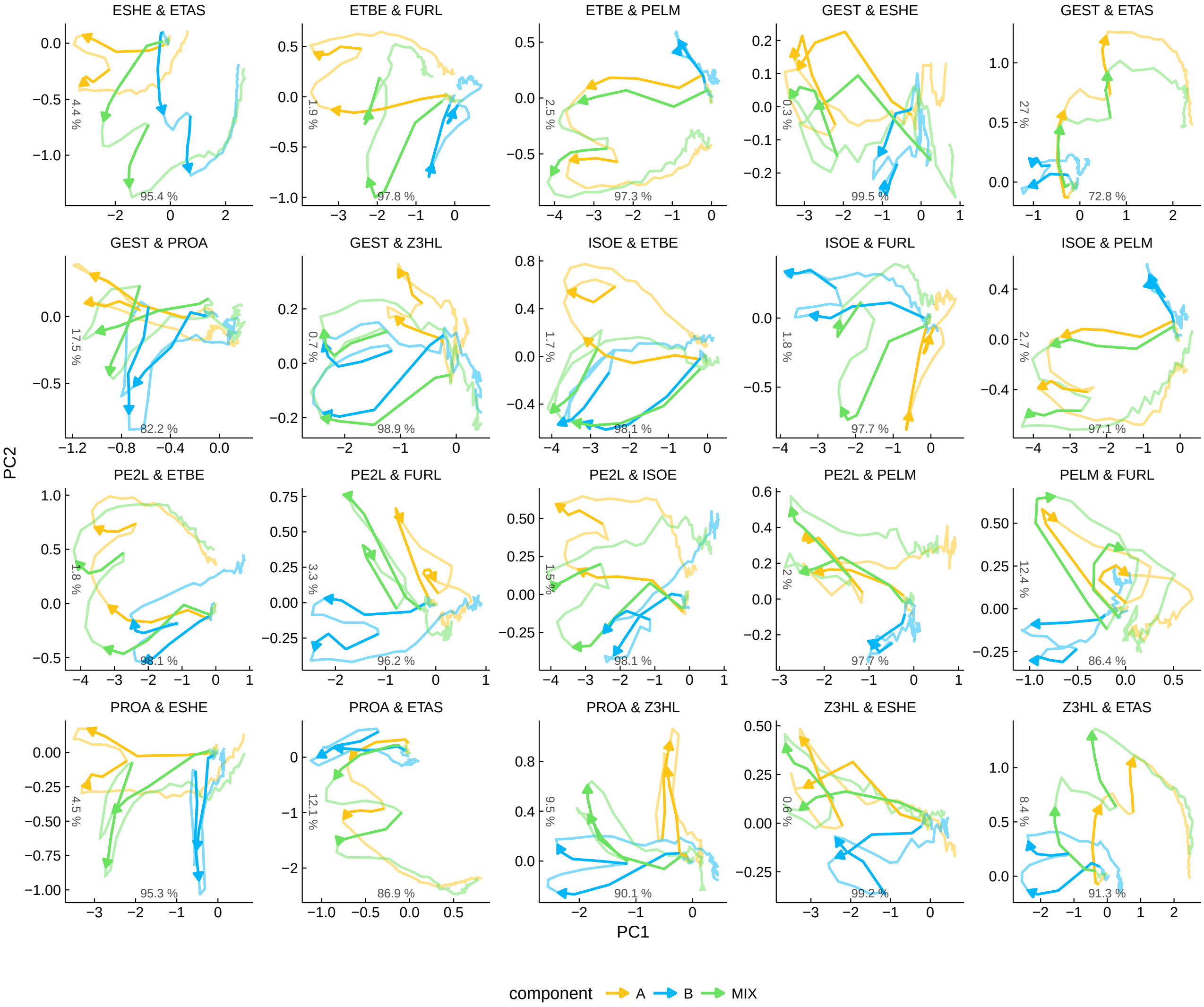

Figure S8 Related to Figure 6. Principal component trajectories of mixture and component responses. Trajectories show how the odor response pattern of the five analyzed OSNs develops over time. Times of odorant stimulation are indicated by darker arrows pointing in the direction of time. Numbers on the axes indicate the percentage of variance explained by the corresponding principal component
